# Elevated methylmercury in Arctic rain and aerosol linked to air-sea exchange of dimethylmercury

**DOI:** 10.1126/sciadv.adr3805

**Published:** 2025-03-19

**Authors:** Yipeng He, Hannah Inman, David C. Kadko, Mark P. Stephens, Douglas E. Hammond, William M. Landing, Robert P. Mason

**Affiliations:** ^1^ Department of Marine Sciences, University of Connecticut, Groton, CT 06340, USA.; ^2^ Applied Research Center, Florida International University, Miami, FL 33174, USA.; ^3^ Department of Earth Sciences, University of Southern California, Los Angeles, CA 90089, USA.; ^4^ Department of Earth, Ocean and Atmospheric Science, Florida State University, Tallahassee, FL 32306, USA.

## Abstract

Mercury (Hg) is a global pollutant with substantial human health impacts. While most studies focus on atmospheric total Hg (THg) deposition, contributions of methylated Hg (MeHg), including monomethylmercury (MMHg) and dimethylmercury (DMHg), remain poorly understood. To examine this, we use rain and aerosol Hg speciation data and high-resolution surface DMHg measurements, collected on a transect from Alaskan coastal waters to the Bering and Chukchi Seas. We observed a significant fivefold increase in the MeHg:THg fraction in rain and a 10-fold increase for aerosols, closely linked to elevated surface DMHg and the highest DMHg evasion (~9.4 picomoles per square meter per hour) found in upwelling waters near the Aleutian Islands. These data highlight a previously underexplored aspect of MeHg air-sea exchange and its importance to Hg cycling and human health concerns. Our findings emphasize the importance of DMHg evasion by demonstrating that atmospheric MeHg can be transported long distances (~1700 kilometers) in the Arctic, posing risks to human health and ecosystems.

## Teaser

Dimethylmercury evasion could elevate methylmercury levels in Arctic rain and aerosols, affecting ecosystems and human health.

## INTRODUCTION

Monomethylmercury (MMHg), an organic form of mercury (Hg) and a bioaccumulative neurotoxin for both wildlife and humans (*1*, *2*), is predominantly formed within aquatic systems and appears to be ubiquitous in all environmental compartments and biota (*3*). Continued anthropogenic Hg emissions have increased Hg levels in the environment where it is methylated to MMHg and then biomagnified throughout aquatic food webs, eventually causing high exposure to humans (*2*, *4*). Globally, MMHg formation is predominantly from inorganic Hg and is mediated by microbes in aquatic systems (*5*, *6*).

Dimethylmercury (DMHg), another toxic organomercurial, is predominantly found in marine environments, such as the deep sea, upwelling regions, and sea ice (*7*–*10*), and also nonmarine environments, such as lakes, mangroves, and saltmarsh sediments (*11*–*13*). Unlike MMHg, DMHg is volatile and can degas into the atmosphere, potentially altering the environmental pool of methylated Hg (MeHg = MMHg + DMHg). There is evidence that DMHg can be formed both directly from inorganic Hg and from MMHg (*14*); however, the mechanisms are still unclear. Some studies have suggested that DMHg transforms into MMHg in the ocean (*15*–*17*); however, the pathways of conversion are still not unequivocally demonstrated. In addition, a recent study found that DMHg could undergo photochemical demethylation to form MMHg (*18*). Nevertheless, the volatility of DMHg opens the possibility of a previously underexamined pathway for MeHg transport through the atmosphere (*19*)—i.e., DMHg, emitted from the surface ocean, is transformed to MMHg in the atmosphere, incorporated into precipitation and aerosols, potentially transported long distances through atmospheric circulation, and ultimately deposited into oceans and to the terrestrial landscape. Thus, if such pathways exist in many locations, then the MeHg exposure risk for wildlife and humans in some regions is not only from its in situ production but also from atmospheric deposition. This pathway needs more attention, given the potential for enhanced MMHg exposure (*19*, *20*). However, there has been little quantification of DMHg evasion from the global oceans or its atmospheric fate, and our understanding of this pathway is insufficient.

Globally, anthropogenic Hg emissions to the atmosphere have increased current atmospheric Hg concentrations by about 450% above natural levels (*21*). These emissions have spread Hg worldwide through atmospheric transport, ocean circulation, and river runoff, leading to an increase in total Hg (THg) concentrations in the oceans (*3*). A recent global Hg budget suggests that atmospheric Hg deposition, as Hg^0^ and Hg^II^, supplies total fluxes to the open ocean and snow/ice of 7220 and 410 Mg year^−1^, respectively (*22*). However, owing to a lack of the MeHg data in rain and aerosols, there are few compilations of the global atmospheric MeHg flux (*2*). Despite this, some studies have provided valuable insights into regional estimates of MeHg flux magnitude (*7*, *23*–*25*). For example, a study in the coastal region in Hudson Bay and the Canadian Arctic Archipelago (CAA) provided the first DMHg measurement in ambient air and suggested that the evasion of DMHg from surface waters can sustain the local atmospheric MMHg concentrations. However, because of no observed MeHg data of rain and aerosol, the conclusion from this regional study was limited (*25*). Recent studies estimating the Arctic Hg budget suggest that atmospheric deposition fluxes for MeHg are 2.6 Mg year^−1^ (*23*), while atmospheric deposition fluxes for THg are 64.5 Mg year^−1^ (*26*), resulting in a MeHg:THg fraction (%MeHg) of 4.0%. This fraction is higher than those measured in rain (1.2 ± 0.64%) and aerosol (0.53 ± 0.5%) samples collected during the Arctic GEOTRACES cruise (*27*). This Arctic budget also suggested that >90% of the DMHg in the polar mixed layer (PML) was lost to the atmosphere, with the rest converted to MMHg (*24*). However, while earlier studies in the CAA found high concentrations of DMHg in surface waters (*14*, *28*, *29*), studies in the open Arctic have generally reported low DMHg in the PML (*8*, *23*). Last, while Schartup *et al.* (*10*) found evidence for DMHg in ice cores, Jonsson *et al.* (*23*) did not. Overall, these studies demonstrate that there is little consistency in the understanding of the sources and cycling of methylated Hg in the Arctic, and this is true for many of the ocean surface waters and atmospheric boundary layer. This lack of understanding of the importance of DMHg evasion motivated the current study.

Overall, there are few measurements of MeHg in precipitation over the open ocean, but the MeHg:THg fraction is typically 1 to 2% (*30*, *31*), except for some coastal regions. For example, in coastal California, fog samples contained a relatively higher MeHg:THg fraction of 5.7% (*32*, *33*). Coastal upwelling locations in California were found to have higher DMHg surface water concentrations (*7*). These findings have led to our hypothesis that DMHg evasion from the surface ocean is a critical determinant of the MeHg level in the atmosphere, especially for rain and aerosols and, as a consequence, of MeHg in wet and dry deposition.

Despite being the smallest of Earth’s oceans, the Arctic Ocean plays a unique role in Hg cycling because of its distinctive physical, chemical, and biological characteristics, which are influenced by the presence of sea ice, permafrost, and glaciers (*26*). Hg inputs to Arctic ecosystems originate from long-range atmospheric transport of global Hg emissions with equally important river runoff and ocean current inputs and with local anthropogenic contributions being negligible (*26*). Despite the limited local anthropogenic input, Hg levels in Arctic biota, such as fish, marine mammals, and seabirds, are significantly higher than those at lower latitudes (*34*) and disproportionately cause higher Hg exposure risk to the Arctic indigenous people because of their hunting and fishing activities (*35*, *36*). Furthermore, with the accelerated warming in polar regions (*37*), Hg exposure risk for Arctic communities and wildlife is anticipated to increase because of intensified Hg inputs. These inputs could be augmented by less ice and more MeHg formation and bioaccumulation, reflecting enhanced Hg mobilization between seawater, sea ice, permafrost, and the atmosphere (*38*–*40*). While the fluxes of THg and MeHg between the Arctic atmosphere and surface ocean have been estimated, the MeHg flux estimate has a large uncertainty because of a lack of observational data. For instance, estimates of total Arctic atmospheric MeHg deposition range from 2.6 to 8.5 Mg year^−1^, while MeHg ocean evasion estimates range from 4.5 to 14 Mg year^−1^ (*23*, *24*). The limited availability of MeHg data in rain and aerosol samples represents a critical gap in understanding the cycling of MeHg in the Arctic. In addition, while there have been some DMHg measurements (*8*, *25*, *41*), because of technological challenges, the number and location of these DMHg measurements at the ocean surface are insufficient, further complicating our understanding of the role of air-sea exchange in regulating MeHg levels in the Arctic Ocean.

To address these knowledge gaps, we initiated an Arctic expedition aimed at investigating the air-sea exchange of inorganic Hg and MeHg and evaluating its significance within the broader context of Hg biogeochemical cycling. We also used a newly developed DMHg autoanalyzer (DAA) (*42*) and conducted, to our knowledge, the first Arctic high-resolution (hourly) DMHg measurements in surface ocean waters while also collecting rain and aerosol samples for determining Hg species. In conjunction, water column measurements were made for all Hg species. Here, we show that DMHg evasion from the surface ocean is not ubiquitous in Arctic waters (AWs), but when it occurs, its evasion is tightly coupled with elevated MeHg levels in rain and aerosols, demonstrating that DMHg evasion can influence atmospheric MeHg deposition over a broad spatial scale.

## RESULTS

### Observations of DMHg evasion from surface ocean

**Fig. 1.**
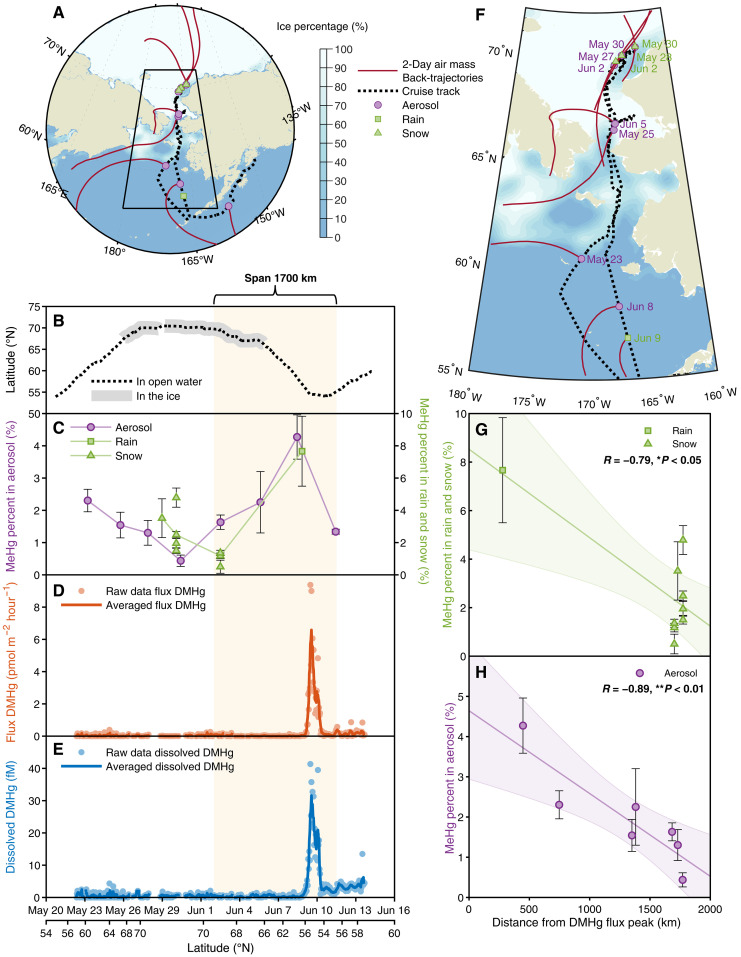
Arctic cruise track with the Arctic sea-ice cover information and results of MeHg percent in rain and aerosol and underway DMHg measurement and its flux. (**A**) Arctic cruise track (black dotted line) overlaid on a map indicating sea-ice cover percent (colored bar, sea-ice percent; National Snow and Ice Data Center). Aerosol deployment midpoints (purple circles), rain sampling location (green square), and snow sampling locations (green triangles) with 2-day air mass back-trajectories (red lines) using NOAA’s HYSPLIT model (*70*) are shown. (**B**) Cruise latitude information (black dotted line) juxtaposed with marginal sea ice and sea-ice zone (gray area) and the estimated span of elevated atmospheric MeHg deposition (light khaki area between 54° and 69°N). (**C**) Measured fractions of MeHg:THg in rain (green square; right axis), snow (green triangles; right axis), and aerosol (purple circles; left axis) from this study (tables S4 to S6). (**D**) Calculated air-sea exchange fluxes of DMHg in surface seawater during the cruise (light orange dots) with a 5-hour moving averaged trend (orange line). (**E**) Dissolved DMHg concentrations measured in surface seawater during the cruise (light blue dots) with a 5-hour moving averaged trend (blue line). Data points are correlated with corresponding date time and latitude. (**F**) A zoom-in view of the cruise track region and same legends as indicated in (A) with purple and green colored dates for the aerosol and rain/snow collection dates, respectively. (**G**) Linear correlation between measured fractions of MeHg:THg in rain (green squares) and snow (green triangles) and the distance from the DMHg flux peak. (**H**) Linear correlation between measured fractions of MeHg:THg in aerosol (purple circles) and the distance from the DMHg flux peak. Robustness is denoted as Pearson’s *R*; the significance level is denoted as **P < 0.01 and *P < 0.05.

Our high-resolution hourly measurements of DMHg in surface seawater, collected in Alaskan coastal waters and across the Bering and Chukchi Seas (from 55° to 70°N), represent the first dataset of its kind for the open ocean. These data highlight the usefulness of high-resolution measurements in resolving ongoing controversies about the air-sea exchange of DMHg in the Arctic. These measurements made using the DAA (*42*) traversed through the open ocean and marginal ice zone and into the sea-ice zone (Fig. 1A). Dissolved DMHg in surface seawater was generally low in the open ocean waters traversing into the sea-ice zone with averages of 2.3 fM [range detection limit (DL), 13.5 fM; *n* = 95], 0.4 fM (range DL, 4.3 fM; *n* = 99), and 0.5 fM (range DL, 3.4 fM; *n* = 148) in the south Bering Sea (55° to 60°N), north Bering Sea (60° to 65°N), and Chukchi Sea, respectively (Fig. 1E and table S2). Our values are comparable to the PML DMHg concentrations of 2.3 ± 1.7 fM found by Jonsson *et al.* (*41*), and we also found low DMHg at the shallowest bottle sampling depth during the cruise (<6 fM; table S7). However, a significantly elevated concentration of dissolved DMHg was observed near the Aleutian Islands in the coastal Alaska region with an average of 10.26 fM (range, 0.9 to 41.4 fM; *n* = 51), which was up to 20 times higher than the average value for the other locations. A high concentration was also found at 10 m at a nearby station (~36 fM; Station 16; table S7).

As there are no previous studies directly measuring dissolved DMHg in the surface open ocean in this region, we summarized our surface measurements of dissolved DMHg and compared them with published surface DMHg data from previous studies, typically collected at 10 m. Globally, surface ocean dissolved DMHg displays considerable variability. For example, the highest dissolved DMHg has been recorded in Monterey Bay, California, which is an upwelling location, with an average of 73.3 ± 86.9 fM (*n* = 15) (*7*) compared to the open ocean, which exhibited lower concentrations, such as in the South Atlantic (5.3 ± 5.5 fM, *n* = 6) (*15*) and central tropical Pacific (9.8 ± 9.7 fM, *n* = 12) (*43*). Similarly, low surface DMHg concentrations were also found in the North Atlantic (*44*) and South Pacific surface waters (*9*). The coastal region of the Arctic in Hudson Bay and CAA also showed higher DMHg (39.6 ± 60.0 fM; *n* = 20) (*25*, *45*) than the open waters of the Arctic. Comparing our measurements with the global data, dissolved DMHg in the Bering Sea and Chukchi Sea was lower than that in the mid- and low-latitude open ocean surface waters, while the dissolved DMHg in the coastal Alaska region was at the higher end of the published data.

Similar to the dissolved DMHg pattern, the DMHg evasion flux was notably low in the south and north Bering Sea and Chukchi Sea with averages of 0.14 pmol m^−2^ hour^−1^ (range, 0 to 0.86 pmol m^−2^ hour^−1^; *n* = 94), 0.026 pmol m^−2^ hour^−1^ (range, 0 to 0.39 pmol m^−2^ hour^−1^; *n* = 99), and 0.029 pmol m^−2^ hour^−1^ (range, 0 to 0.26 pmol m^−2^ hour^−1^; *n* = 148), respectively (see table S2). The flux was, however, significantly higher in the coastal Alaska region with an average of 1.59 pmol m^−2^ hour^−1^ (range, 0.014 to 9.38 pmol m^−2^ hour^−1^; *n* = 51; Tukey’s post hoc test, *P* < 0.001; Fig. 1D and table S2), which was comparable to the DMHg flux measured in the coastal regions in Hudson Bay and the CAA (*45*). Furthermore, the differences in fluxes between ocean regions in this study are primarily driven by variations in DMHg concentrations rather than differences in wind speed and temperature, which are also major factors influencing gas exchange.

**Fig. 2.**
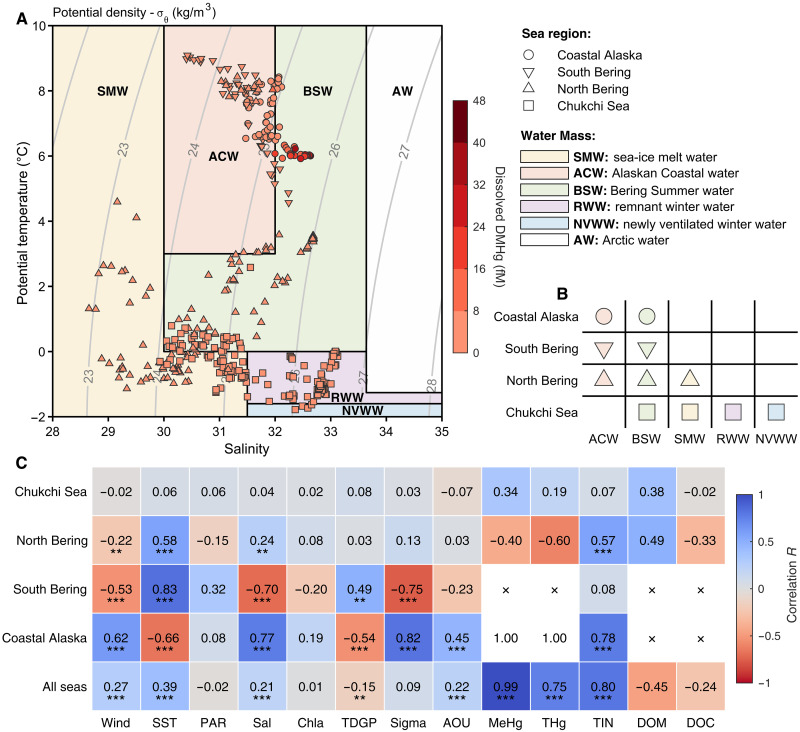
Distribution and correlation of dissolved DMHg with environmental parameters in various water masses during the cruise. (**A**) Spatial distribution of sampled underway DMHg and its concentration (colored bar) across different water masses, categorized by potential temperature and salinity properties. Water masses are divided into SMW (light yellow), ACW (light pink), BSW (light green), RWW (light purple), NVWW (light blue), and AW (white) with the contours of potential density overlaid (gray) among coastal Alaska (circles), South Bering (downward triangles), North Bering (upward triangles), and Chukchi Sea (squares) (*47*). The water mass boundaries are indicated by the black lines. (**B**) Distribution of water masses in different region of oceans during the cruise. (**C**) Correlation analysis on DMHg concentrations, water mass properties, and environmental parameters, represented by Pearson’s correlation coefficients (colored bar), including wind speed (Wind), SST, photosynthetically active radiation (PAR), salinity (Sal), chlorophyll a (Chla), total dissolved gas pressure (TDGP), potential density (Sigma), AOU, MeHg, THg, TIN, dissolved organic matter (DOM), and dissolved organic carbon (DOC). Significance levels are denoted as ***P < 0.001, **P < 0.01, and *P < 0.05. The water mass properties and environmental parameters associated with this cruise are available in fig. S2.

To further investigate the sources and distribution of surface DMHg, we conducted a water mass analysis, classifying the water masses on the basis of their potential temperature and salinity (*46*). In the Bering Sea and Chukchi Sea, surface water masses can be categorized as sea-ice melt water (SMW), Alaskan coastal water (ACW), Bering summer water (BSW), remnant winter water (RWW), newly ventilated winter water (NVWW), and AW (*46*, *47*). Notably, the highest dissolved DMHg was in the BSW, especially in the coastal Alaska region, which was also a major water mass component in the Bering Sea and Chukchi Sea (Fig. 2, A and B). BSW forms at the beginning of the Arctic summer, originating from the Bering Shelf with high salinity, and is then transported northward through the Bering Strait into the Chukchi Sea over the course of 1 to 2 months (*47*). Therefore, there is a potential pathway for this water mass, containing the high DMHg, to be transported northward through the Bering Sea and into the Chukchi Sea. However, because of the distance and the unstable chemistry of DMHg in surface AWs (*18*), DMHg would start to degrade after leaving the source region, leading to a gradually decreasing concentration from the Bering Sea to Chukchi Sea. In addition, DMHg evasion during the northward transport of BSW would further contribute to DMHg loss.

Correlation analyses of DMHg and other variables suggest that, in the high DMHg coastal Alaska region, its surface concentration is positively correlated with wind intensity (Pearson’s coefficient *R* = 0.62, *t* test, *P* < 0.001) and negatively correlated with sea surface temperature (SST; Pearson’s coefficient *R* = −0.66, *t* test, *P* < 0.001), implicating a role for wind-driven coastal upwelling (Fig. 2C). Moreover, positive correlations were also observed with apparent oxygen utilization (AOU; Pearson’s coefficient *R* = 0.45, *t* test, *P* < 0.001) and total inorganic nitrate (TIN; Pearson’s coefficient *R* = 0.78, *t* test, *P* < 0.001), suggesting the involvement of both upwelling and biological processes in DMHg production (Fig. 2C). Higher Hg methylation potential in the coastal Alaska seawater has been linked to microorganism abundance and nitrification activity (*48*). Furthermore, in the highly productive upwelling California Current System (*17*), elevated dissolved DMHg and THg concentrations were observed in the upwelled water mass, while increased MMHg was detected after the water mass advected away. This highlights the potential for DMHg formation in deep waters, demethylation in surface waters, and the possible sustainment of a larger MeHg pool in this upwelling region. Overall, DMHg on the Bering Sea shelf appears to be formed in deeper waters, possibly upwelled into BSW near the Aleutian Islands in the coastal Alaska region, and then potentially transported northward. However, given the limited reported data for DMHg in surface waters, we cannot extrapolate our results globally concerning DMHg cycling, and the mechanisms of its formation and degradation in the ocean are still unclear.

### Hg species in wet and dry deposition

**Fig. 3.**
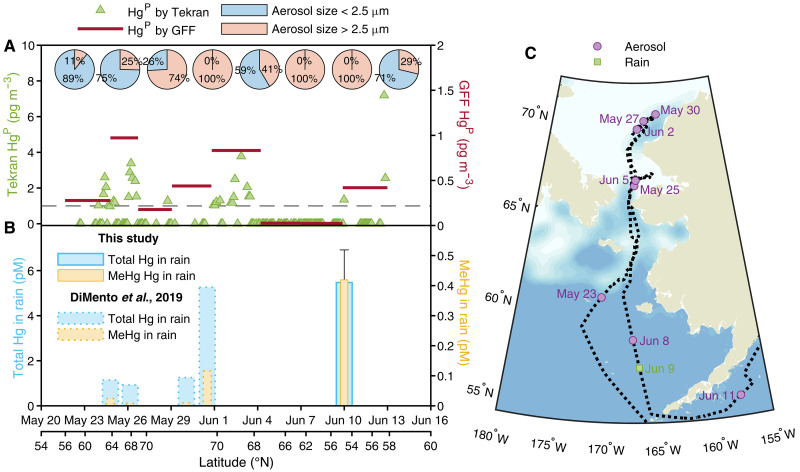
Mercury speciation in size-fractionated aerosol and rain. (**A**) Tekran speciation system measurements of THg in aerosols <2.5 μm (green triangles, left axis) compared with THg in a high-volume bulk aerosol sampler for pan-size aerosols on the GFF (red bars represent the sampling duration; right axis). The pie chart shows the distribution of THg in aerosols, divided into <2.5-μm (blue sector) and >2.5-μm (pink sector) size fractions. The dashed gray line denotes the DL of the THg in aerosol by the Tekran speciation system. (**B**) Measured concentrations of THg (blue bars) and MeHg (yellow bars) in rain from this study (solid outline bars) contrasted with the US Arctic GEOTRACES cruise (*27*) (dotted line bar; see table S5). Data points are correlated with corresponding date time and latitude. (**C**) A zoom-in view of the Arctic cruise track (black dotted line) overlaid on a map indicating sea-ice cover percent (colored bar, sea-ice cover percentage; National Snow and Ice Data Center) reflecting sea-ice conditions during the cruise. Aerosol deployment midpoints (purple circles) and rain sampling location (green square) with purple and green colored dates for the aerosol and rain collection date, respectively.

The wet and dry deposition fluxes of Hg species are fundamentally determined by the presence of these species in the atmosphere, either in gaseous form or attached to aerosols. A series of bulk aerosol samples were collected using a high-volume sampler, as done on previous GEOTRACES cruises (*27*, *30*). The Tekran speciation system (1130/1135/2357X) was also deployed to collect high-resolution particulate Hg (Hg^P^) in the smaller-size aerosol (<2.5 μm), given the size cutoff at the Tekran sample inlet for larger aerosols (*49*). The bulk Hg^P^ levels were generally low compared to land-based Arctic observations (*50*) but consistent with previous observations from the 2015 Arctic GEOTRACES cruise (*27*), with an average of 0.40 pg m^−3^ (range, 0.02 to 0.97 pg m^−3^; *n* = 8; see table S4). The lowest Hg^P^ levels were recorded during 4 to 10 June (Fig. 3A), coinciding with snow and rain events that scavenged aerosols from the atmosphere, depositing them onto sea ice and the surface ocean. For the aerosol size <2.5 μm, most of the data were below the DL of the Tekran speciation system [DL, 1 pg m^−3^; (*49*)]. There were, however, a few higher Hg^P^ measurements (see table S3), possibly due to factors like wind-induced sea spray or different air masses. Nevertheless, the overall pattern of bulk Hg^P^ aligned with the Tekran Hg^P^ measurements. The comparison of the two datasets indicated that the smaller-size aerosols were dominant in the south Bering Sea (Fig. 3A), suggesting that they could be derived from the land, while larger-size aerosols were likely associated with sea spray. Intriguingly, the MeHg levels in the aerosol filters (MMHg^P^) mostly exceeded the values observed during the 2015 cruise (*27*), with an average of 5.8 fg m^−3^ (range, 0.6 to 15 fg m^−3^; *n* = 8; see table S4), indicating interannual variability in MeHg levels in Arctic aerosols. On the other hand, rainwater THg was 5.5 ± 1.4 pM, comparable to the previous study (*27*) (range, 0.9 to 5.2 pM; Fig. 3B and table S5). Similarly, surface snow, collected on sea ice, THg was 6.4 ± 4.8 pM (table S6), comparable to the previous study (range, 1.9 to 6.0 pM) (*27*). However, the MeHg level in the rainwater and snow was notably higher at 0.42 and 0.12 ± 0.06 pM, respectively, surpassing previous measurements of Arctic rain and snow in the vicinity of the Chukchi Sea (Tukey’s post hoc test, *P* < 0.001 for both rain and snow) (*27*).

**Fig. 4.**
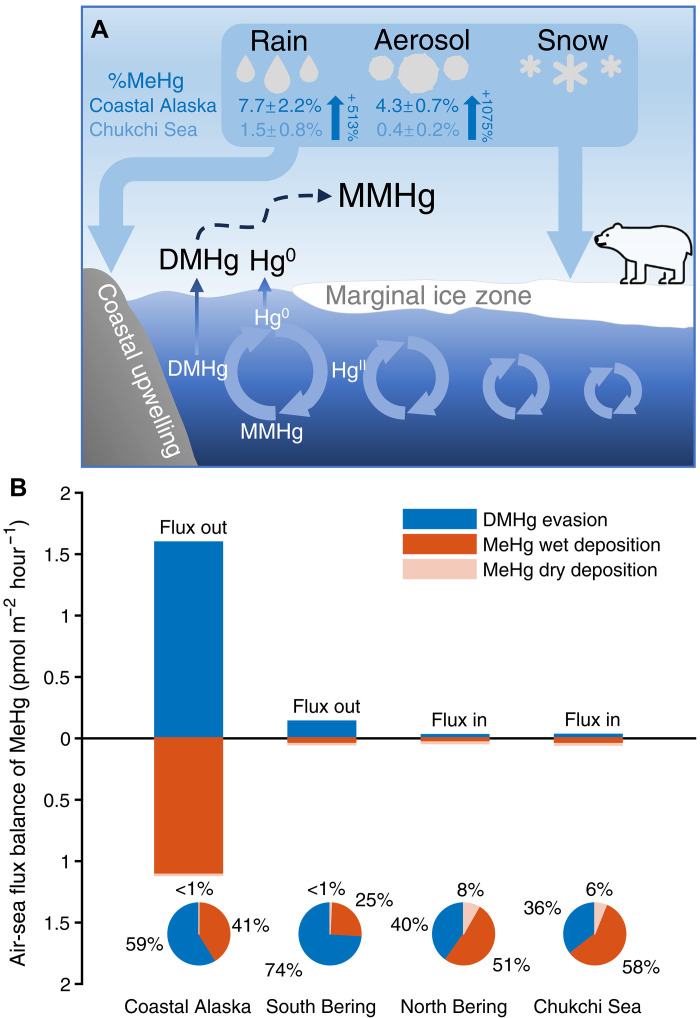
Schematic diagram of the understanding of DMHg evasion from the surface ocean to the atmosphere and the balance of MeHg fluxes in the Arctic marine boundary layer. (**A**) Elevated methylated Hg level in rain and aerosol with their MeHg fractions from the Coastal Alaska (dark blue) and Chukchi Sea (light blue) transport and deposit to a large area in the Arctic, demonstrating the impact of the air-sea exchange of DMHg on the regional mercury biogeochemical cycle. (**B**) Balance between DMHg evasion (blue bar) and atmospheric deposition of MeHg, both wet (dark orange bars) and dry (light orange bars), in the coastal Alaska region, south Bering Sea, north Bering Sea, and Chukchi Sea. A pie chart details the relative magnitudes and ratios of these fluxes in each region. “Flux out” represents the net MeHg flux leaving the surface ocean, while “Flux in” indicates the net influx of MeHg into the surface ocean.

Furthermore, while the MeHg:THg fraction in aerosols was lower in the Chukchi Sea near 70°N, ~0.4 ± 0.2%, resembling the MeHg:THg fraction observed in aerosols (0.53 ± 0.50%) previously (*27*), it was 10 times higher further south at 4.3 ± 0.7% (calculated relative to the lowest value of the MeHg:THg fraction in aerosols in the Chukchi Sea). In addition, the MeHg:THg fraction in rainwater was five times higher than the averaged fractions (1.5 ± 0.8%) from the 2015 Arctic GEOTRACES cruise (*27*), being about 7.7 ± 2.2% in the coastal Alaska region (Fig. 4A). Trend analyses of the MeHg:THg fraction in both rain/snow and aerosols were compiled on the basis of the distance between the DMHg flux peak location and these sampling locations (Fig. 1, G and H). Unexpectedly, two decreasing trends of MeHg:THg fraction in both rain/snow and aerosols were apparent with the increased distance from the Bering Sea to the Chukchi Sea in 2021 (Fig. 1G for rain/snow, Pearson’s coefficient *R* = −0.79, *t* test, *P* < 0.05; Fig. 1H for aerosols, Pearson’s coefficient *R* = −0.89, *t* test, *P* < 0.01), with the maxima approaching simultaneously at about 55°N near the Aleutian Islands. While the decreasing MeHg:THg trend in rain and snow is driven by only one high data point collected during the cruise, a more robust decreasing trend was found (fig. S3, Pearson’s coefficient *R* = −0.68, *t* test, *P* = 0.01) by combining previously collected rain data from the Chukchi Sea (*27*). The maximum value coincides with the peak of surface water DMHg concentration and evasion (Fig. 1, D and E). In addition, the observed maximum MeHg:THg fraction likely reflects the transformation of DMHg into MMHg, which is subsequently adsorbed onto or incorporated into aerosols and rain. As the air mass moves northward, the decreasing MeHg fractions in aerosols and rain may result from mixing with other air masses containing lower MeHg levels or from MeHg demethylation occurring in the atmosphere. This synchronicity of events demonstrates a previously undocumented aspect of DMHg evasion, connecting it to the atmospheric MeHg pool and raising important questions about how and why the elevated MeHg:THg fractions in rain and aerosols are closely linked to the observed DMHg flux.

## DISCUSSION

### Insight into DMHg evasion and its global impact

Globally, measurements of DMHg evasion from the surface ocean are exceedingly rare. This scarcity of data primarily results from the analytical challenges and sampling constraints associated with the trace concentrations of DMHg (*51*) and its propensity for demethylation in seawater (*18*). As a consequence, understanding how DMHg evasion influences atmospheric MeHg levels has remained an enigmatic challenge. Given this knowledge gap, we evaluated several previous studies (*25*, *28*, *29*, *32*, *45*, *52*) and derived three possible hypotheses here to explain the observed MeHg in deposition: (I) the elevated MeHg in the aerosols and rain mainly comes from ocean MMHg evasion; (II) the elevated MeHg is caused by atmospheric Hg^II^ methylation; and (III) the elevated MeHg is related to the DMHg evasion and its subsequent degradation to MMHg, which could result in a large spatial influence on MeHg deposition.

Hypothesis I treats the surface ocean MMHg as the major source to the atmospheric MeHg pool by its gas evasion. However, because of the higher solubility and lower volatility of MMHg (gas-aqueous distribution constant, *H* = 1.9 × 10^−5^ at 25°C for CH_3_HgCl) compared to DMHg (gas-aqueous distribution constant, *H* = 0.31 at 25°C) in seawater (*53*, *54*) and its low surface seawater concentrations (*8*, *23*), the possibility of generating gaseous atmospheric MMHg through exchange at the air-sea microlayer is much lower than for DMHg. Another proposed mechanism for MMHg evasion is through sea spray, which can subsequently be absorbed into the aerosols or scavenged by precipitation. However, most observed surface seawater MeHg:THg fractions fall below the highest fraction found in aerosol (4.3%) or rain (7.7%) in this study. Therefore, this hypothesis is an untenable explanation for the data reported here, given MeHg:THg fractions measured in surface waters in this region (*8*, *23*). In addition, ratios of MeHg level between rain and aerosols observed in this study are much higher than reported in other studies (*19*, *27*, *32*, *33*), suggesting that surface aerosols, such as sea salt, are not the major contributor to the MeHg in rain.

Hypothesis II requires the support of in situ Hg methylation in aerosols and/or in precipitation. A previous study has explored the possibility for abiotic Hg methylation in natural waters by adding acetic acid (CH_3_COOH) or acetone (CH_3_COCH_3_) as a methyl group donor (*52*). However, the rates of methylation found in these laboratory studies exceed, by two orders of magnitude, the rates of methylation found to occur biotically in seawater and freshwater (*14*, *28*), suggesting that these rates are not realistic. If they were, then abiotic methylation would be more important than biotic methylation in natural waters, and no studies have demonstrated this to occur. Hammerschmidt *et al.* (*55*) concurred in their calculations that abiotic processes could only account for the measured ratios of MMHg:Hg^II^ in rain under conditions with unrealistic abiotic methylation rates. In addition, there would be competing abiotic demethylation reactions (photodegradation of MMHg to Hg^II^) that would reduce the net MMHg production. Now, there is no evidence for the biotic Hg methylation in rain and/or aerosols mediated by microbial activity. Therefore, this hypothesis is implausible based on the current understanding.

Hypothesis III posits that the elevated MMHg in aerosols and rain is primarily caused by DMHg evasion from the surface ocean and is the most likely explanation for the results of our study. While prior studies have suggested a link between the DMHg evasion and the atmospheric MMHg pool (*19*, *25*, *32*), direct observational evidence from simultaneously observing DMHg evasion and measuring the MeHg level in rainwater and aerosols was lacking before our study. Our high-resolution DMHg evasion flux data match with the atmospheric MeHg pool pattern and the variability in the MeHg:THg fraction over the cruise duration, explaining the MMHg:THg fraction variability in the aerosol and possibly in rain. Furthermore, using our data, we have compared the dry and wet deposition fluxes of MMHg to those of DMHg evasion, accounting for aerosol dry deposition rates and Arctic spring precipitation [10-year average from ERA5 (*56*)]. In the coastal Alaska region and south Bering Sea, the DMHg evasion flux exceeds MMHg dry and wet deposition fluxes by factors of 1.4 and 2.8, respectively (Fig. 4B and table S8), supporting the notion that, in these regions, DMHg evasion could sustain the atmospheric MMHg pool and its deposition. However, in the north Bering Sea and Chukchi Sea, where the DMHg flux is much smaller and lower than MMHg deposition fluxes (Fig. 4B and table S8), an additional MMHg supply is required to balance the net flux. Elevated MMHg:THg fractions in aerosols were observed not only in the coastal Alaska region, where the DMHg concentration reached its peak, but also in the Bering Sea and Chukchi Sea over a distance of 1700 km from the peak DMHg evasion region. Given the prevailing northward winds (fig. S9) and the air mass back-trajectory analysis (Fig. 1A), we conclude that DMHg evasion from the coastal Alaska region is indirectly influencing the atmospheric MMHg pool to the north. Therefore, the proposed transformation process involves DMHg being transformed into MMHg by gas-phase reactions (*57*–*59*), which is subsequently adsorbed onto or incorporated into aerosols and rain, or by reactions in fog or clouds (*33*), thereby increasing their MeHg concentrations. These MeHg-enriched aerosols and precipitation can then be deposited onto the surface ocean, sea ice, and terrestrial environments (Fig. 4A).

Our findings underscore the pivotal role of DMHg evasion at the ocean surface, establishing a close link between atmospheric and marine MeHg dynamics, both locally and remotely. These dynamics are of critical importance as elevated atmospheric MeHg deposition directly contributes to the ocean MeHg pool available for accumulation into the food web. On a global basis, atmospheric MMHg may not be the major flux to the surface ocean, but our data indicate its regional importance. In the Arctic Ocean, atmospheric deposition of MeHg is estimated at 2.6 Mg year^−1^, which is three times greater than the riverine export of MeHg (0.8 Mg year^−1^) to the region. The surface ocean (0 to 20 m) and the entire mass of sea ice contain ~4 and 0.32 Mg of MeHg, respectively (*23*). Consequently, MeHg exposure risk for wildlife and humans in specific regions results not only from MeHg produced within the ocean but also from atmospheric deposition, suggesting the need for a thorough re-evaluation of the current methylated Hg cycle in the surface ocean and boundary layer to improve MeHg exposure risk assessments. Driscoll *et al.* (*2*) concluded that global deposition of MMHg was five times the evasion flux. In contrast, while a more recent ocean model of methylated Hg dynamics (*60*) included a much larger flux of DMHg, this model did not include deposition of MMHg to the surface ocean and concluded that the water column demethylation flux for DMHg was smaller than for MMHg in the surface ocean and excluded its photodemethylation, which has been shown to be similar to that of MMHg (*18*, *61*). Our data for the Arctic indicate differing behavior for this region. Additional work is needed to clarify how widespread this behavior is and to support more accurate modeling of methylated Hg cycling and the dynamics of atmosphere-ocean exchange.

Furthermore, because this study demonstrates that gas exchange of DMHg is one important mechanism controlling the MeHg pool in the atmosphere, its concentration is likely to be more dynamic and uncertain, given the current and future climate change. The global oceans are experiencing higher surface temperature, intensified surface upwelling, and increased primary production (*62*), which may significantly affect DMHg surface water concentrations and its evasion flux, as well as the transport and deposition of MeHg to ocean regions remote from the sources of DMHg, thereby exacerbating MeHg bioaccumulation in marine ecosystems. Specifically, for the Arctic with magnified warming, more Hg release from thawing permafrost into the aquatic system could further perturb the MeHg pool and its bioaccumulation (*26*, *39*). Again, future studies of methylated Hg cycling in the surface ocean clearly need to assess the inputs of MMHg from the atmosphere and the evasion of DMHg in more ocean regions.

## MATERIALS AND METHODS

All sampling equipment preparation and sample handling for Hg samples were conducted inside the shipboard ultraclean “bubble” inflated with clean High-Efficiency Particulate Air-filtered air, following the trace metal sampling and handling protocols established by the GEOTRACES Program to minimize potential contamination risks associated with trace metal Hg species (*63*). This unwavering commitment to maintaining the integrity of our samples was essential for producing reliable data and advancing our understanding of Hg dynamics in the studied ecosystems.

### Cruise information

The Arctic Ocean cruise was conducted in coastal Alaskan waters, the Bering Sea, and the Chukchi Sea from 55° to 70°N and back to 55°N through open ocean, marginal ice zone, and sea-ice zone. The R/V *Sikuliaq* departed from Dutch Harbor, Alaska, on 20 May 2021 and sailed north through the Bering Sea, crossing the Bering Strait into the Chukchi Sea, spending more than 2 weeks in the marginal ice zone and sea-ice zone, then sailing south back to Seward, Alaska, and arriving on 14 June 2021 (Fig. 1A). During the cruise, eight aerosol samples and one rain sample were collected and underway dissolved DMHg was measured continuously. In addition, the R/V *Sikuliaq* collected a large amount of monitored underway data on atmospheric and seawater parameters using shipboard instruments (https://www.sikuliaq.alaska.edu/ops/skq_equipment_instrumentation.html).

### Underway dissolved DMHg measurements

To measure dissolved gaseous DMHg in seawater, a continuous counter-flow gas equilibrator chamber was coupled with our newly developed DAA (*42*). This innovative approach, akin to methods used in studying dissolved gaseous Hg^0^ (DGHg), involved drawing ship’s seawater through the chamber via the ship’s intake at a depth of 6 m (*64*–*66*). Briefly, the counter-flow principle of Hg-free air bubbles and water was used to establish continuous equilibrium for gaseous Hg species between the aqueous and gaseous phases. This equilibrium was maintained by exchanging DMHg from the water into counter-flowing air, which was introduced as bubbles through sparging. Before entering the DAA, the gaseous DMHg in the air, equilibrated with the aqueous phase, was thoroughly dried by a soda-lime trap. The measured DMHg concentration in the outgoing gas was equivalent to the dissolved DMHg concentration multiplied by the dimensionless Henry’s law constant specific to DMHg at the prevailing temperature and salinity of the water, as per the following equation

$$\text{DMH}g_{\text{diss}}=\frac{\text{DMH}g_{\text{air}}}{H_{\text{DMHg}}}$$ (1)

where $\text{DMH}g_{\text{air}}$ represents the corresponding DMHg concentration in the outgoing air from the sparger, and $H_{\text{DMHg}}$ is the dimensionless Henry’s law constant for DMHg. The Henry’s law constant is temperature dependent, with detailed calculations available in table S1. The estimation of gas exchange flux was estimated using the following equation

$$F_{\text{DMHg}}=k_{w,\text{DMHg}}\left(\text{DMH}g_{\text{diss}}-\frac{C_{\text{air},\text{DMHg}}}{H_{\text{DMHg}}}\right)$$ (2)

where $C_{\text{air},\text{DMHg}}$ represents the corresponding DMHg concentrations in the ambient atmosphere, and $k_{w,\text{DMHg}}$ is the mass transfer coefficient for DMHg. The mass transfer coefficient is both temperature and wind speed dependent, and again, the detailed calculation of their values for DMHg can be found in table S1. The estimated DLs for DMHg by the DAA in the atmosphere and surface seawater were 5 pg m^−3^ and 0.1 fM, respectively. Calibration of the DAA has been implemented in two ways: (i) calibrating it with known amounts of DMHg vapor before the cruise, requiring careful handling within an evacuated fume hood, and (ii) calibrating it with known amounts of Hg^0^ vapor during the cruise by switching the Carbotrap to a gold trap. More details can be found in (*42*).

### Aerosol filter collection

A high-volume aerosol sampler (TISCH, TE-5170V-BL) was positioned on the front rail of the 03 deck of the ship, ~10 m above sea level, to minimize interference from the ship’s exhaust stack (see fig. S1). The sampler with a similar setup was also used on the 2015 Arctic GEOTRACES cruise (GN01), as well as on the GP16 and GA03 cruises (*27*, *30*). Using procedures to minimize trace element contamination, aerosol samples were collected on glass fiber filters (GFFs; 47-mm diameter, Whatman), which had been baked at 450°C for 12 hours and stored wrapped in foil to prevent any Hg contamination during handling. The GFFs were loaded into Teflon filter holders before each deployment. The Teflon filter holders were cleaned with 0.1 M HCl (trace metal grade purity, Fisher Chemical, Fisher Scientific Co.) and rinsed with deionized water before the cruise. The aerosol sampler operation was controlled on the basis of the wind sector (±60° relative to the ship’s bow) and wind speed (>0.5 m s^−1^) using an anemometer interfaced with a Campbell Scientific CR800 datalogger to prevent contamination from the ship’s exhaust stack. The average sampling duration was ~45.9 hours (31.8 to 61.0 hours) with an average volume per filter of 379 m^3^ (266 to 503 m^3^). After each collection, aerosol filters were transferred to polystyrene petri dishes and double bagged in the ultraclean “bubble.” Blank filters were generated following the same procedures, except that they were not loaded onto the aerosol sampler. All aerosol filters were kept in a dark freezer (−20°C) after collection.

### Tekran speciation system

Measurements of Hg speciation in the near-surface atmosphere were conducted by using the Tekran speciation system (2537B/1130/1135; Tekran Inc.) for determination of Hg^0^, RGHg, and Hg^P^ (*49*), which was also used on the 2015 US Arctic GEOTRACES cruise (*27*), the 2002 Intergovernmental Oceanographic Commission cruise (*67*), and the 2018 US Pacific GEOTRACES cruise (*68*). In this study, our focus was on reporting the measured Hg^P^ concentrations. The Tekran speciation system was deployed on the front rail of the 03 deck of the ship at a height of ~10 m above sea level, side by side with the high-volume aerosol sampler to minimize the influence from the stack (fig. S1). We initiated instrument manual injection calibration using the Tekran 2505 calibration unit while in port, which was repeated to verify and maintain calibration on the Tekran 2537B, with a consistent recovery rate of 98 ± 5%. In addition, the internal permeation source calibrations of Tekran 2537B were performed every 25 hours, so it was calibrated at different times every day. The DLs for the Tekran speciation system were estimated at 1.0 pg m^−3^ for Hg^P^ (*49*, *69*). The Tekran speciation system operated with a standard sampling time of 1 hour for all species, coupled with a desorption time of 1 hour for RGHg and Hg^P^. Consequently, each complete sampling cycle had a duration of 2 hours. Ambient air was drawn through the inlet, initially passing through a heated impactor to remove coarse particles (>2.5 μm). RGHg and Hg^P^ were subsequently absorbed, first onto the KCl-coated denuder and then onto the particulate trap of the Tekran 1130/1135, respectively. Simultaneously, Hg^0^ was quantified using a Tekran 2537B at a time resolution of 5 min. After a 1-hour sampling period, the pyrolyzer, particulate trap, and denuder were heated to 800°, 800°, and 500°C, respectively, while zero air was pumped into the system. This process thermally desorbed and decomposed RGHg and Hg^P^, converting them into Hg^0^, which was subsequently analyzed by the Tekran 2537B. To ensure data integrity and eliminate potential contamination from the ship’s exhaust, we implemented a data validation procedure. This procedure excluded data corresponding to specific conditions: (i) when the ship was stationary at the vertical profile stations during the transect and (ii) when the wind direction was not within ±60° relative to the ship’s bow, adhering to the same operational criteria as the high-volume aerosol sampler.

### Rain sample collection

An automated N-CON precipitation sampler (model GS 00-125, designated for Hg analysis, N-CON Systems Co., Inc.), mounted on the front rail of the 03 deck of the ship at a height of ~10 m above sea level (fig. S1), was used to collect rain (falling snow was not collected for Hg analysis because of its low collection efficiency, given the high relative wind speed when the ship was moving) during the cruise. The sampler was also used on the Arctic GEOTRACES cruise (*27*) and GP16 and GA03 cruises (*30*). It was arranged and operated to avoid contamination from the ship and sea spray by opening for collection only during a precipitation event. Furthermore, the sampler was not operated under high-wind conditions to minimize the collection of sea spray. The rain sampler was fitted with a glass collection funnel, attached to a 1000-ml Teflon sample bottle by a secure Teflon collar placed inside the rain sampler chamber to hold the sampling apparatus in place. To maintain sample integrity, the glass funnel and Teflon sample bottle assemblage were replaced on a weekly basis, even if no rain event had occurred, thereby preventing the accumulation of potential contaminants. The glass funnel and Teflon sample bottle assemblage were cleaned with 0.1 M HCl (trace metal grade purity, Fisher Chemical, Fisher Scientific Co.) and rinsed with deionized water before the cruise. Unfiltered rainwater was collected and transferred to a 100-ml Teflon bottle and acidified to 0.5% (v/v) with sulfuric acid (H_2_SO_4_; trace metal grade purity, Fisher Chemical, Fisher Scientific Co.) and kept in a dark fridge (4°C) for the Hg species analysis.

### Snow sample collection

Snow samples were collected only during the field campaign over sea ice, not during the cruise, as the N-CON precipitation sampler had a low collection efficiency for falling snow, given the high relative wind speed when the ship was moving. Once the ship was nearby the sea ice, it was positioned downwind of the sea ice to prevent chimney smoke and soot from contaminating the surface sea ice. Scientists, along with all research sampling tools, were craned onto the sea ice for the field campaign ~500 m away from the ship. The snow sampling task was first implemented among other tasks to avoid the potential contamination from ship’s exhaust. Fresh surface snow samples were collected into Teflon jars to represent the most recent snow events at each field campaign location. The collected snow samples were stored and thawed into snow water in a dark fridge (4°C). Then, unfiltered snow water was transferred to a 100-ml Teflon bottle and acidified to 0.5% (v/v) with sulfuric acid (H_2_SO_4_; trace metal grade purity, Fisher Chemical, Fisher Scientific Co.) and kept in a dark fridge (4°C) for the Hg species analysis.

### HYSPLIT air mass trajectory

The Hybrid Single-Particle Lagrangian Integrated Trajectory (HYSPLIT) transport model, developed by the US National Atmospheric and Oceanic Administration (NOAA) Air Resources Laboratory, calculates air mass back-trajectories with high-temporal-resolution (every 4 hours) from meteorological data provided by the Global Data Assimilation System interpolated from a 1-by-1° grid in latitude and longitude (*70*). HYSPLIT model runs were performed for ending heights of 50, 100, and 200 m above sea level from the midpoint of each aerosol deployment and the location of rain and snow samples. The cruise track along with the midpoint of each aerosol deployment and each rain and snow sample were overlaid with 2-day air mass back-trajectories to indicate the potential aerosol, rain, and snow source regions collected on this cruise.

### MeHg and THg analysis of aerosol, rain, and snow samples

Aerosol filters were digested in acid-cleaned 15-ml centrifuge tubes (Fisherbrand, Fisher Scientific Co.) with 4.5 M nitric acid (HNO_3_; trace metal grade purity, Fisher Chemical, Fisher Scientific Co.) in a 60°C water bath overnight (*27*). A subsample of this digestate was taken for aerosol MeHg analysis, and the remaining portion was further digested with bromine monochloride (BrCl) at room temperature overnight to convert all Hg species to Hg^II^ for aerosol THg analysis. The method’s DLs for aerosol filters were 20 fg m^−3^ for THg and 1 fg m^−3^ for MeHg based on the average volume of air filtered and the volume of digest analyzed.

Rain/snow samples were also digested overnight, before analysis, in 1% H_2_SO_4_ (trace metal grade purity, Fisher Chemical, Fisher Scientific Co.) for rain/snow MeHg analysis, and a subsample of this digestate was further digested with BrCl at room temperature overnight to convert all Hg species to Hg^II^ for rain/snow THg analysis. The estimated DLs for MeHg and THg in rain/snow were 0.01 and 0.25 pM, respectively.

MeHg concentrations were determined using the direct ethylation method (*71*) with a Tekran 2700 instrument and autosampler, which automated the purging, trapping, and detection through cold vapor atomic fluorescence spectroscopy. Digested aerosol filters and rain samples were neutralized to pH 4.9 with potassium hydroxide (KOH), buffered with acetate buffer, and lastly ethylated with sodium tetraethylborate (NaTEB) to facilitate MeHg analysis. System background MeHg levels were monitored for each analytical run by analyzing solutions with pure reagents in the same vials for each batch of five samples, all analyzed in triplicate. The precision and recovery were determined by analyzing MeHg standards before and after each batch. Sample concentrations were calculated against a standard curve and corrected by operation/spike recoveries, which averaged 103% with a typical relative standard deviation of 7%.

Total mercury concentrations were determined by dual gold amalgamation cold vapor atomic fluorescence spectroscopy using a Tekran 2600 instrument following US EPA Method 1631 (*72*–*74*). Briefly, BrCl-digested aerosol filters and rain samples were pre-reduced with hydroxylamine hydrochloride (NH_2_OH·HCl) to neutralize excess BrCl and then further reduced with stannous chloride (SnCl_2_) to convert all Hg^II^ to Hg^0^ before automated analysis on the Tekran system. System background THg levels were checked for each analytical run by analyzing solutions with only pure reagents in the same vials for each batch of five samples, analyzed in triplicate. Precision and recovery were determined by analyzing THg standards before and after each batch. Sample concentrations were calculated against a standard curve and corrected by operation/spike recoveries with an average of 98 ± 9%.

All analyses were finished within a 3-month period in the dedicated clean lab at the University of Connecticut after the completion of the cruise, ensuring the integrity and accuracy of the collected data.

### Procedural blanks and analysis uncertainty

Procedural blanks for aerosol filters and rain samples were conducted for both MeHg and THg analyses. These blanks encompassed all laboratory procedures, including manipulations, cleaning protocols, and reagent additions, and were subsequently subtracted from the final results to ensure accuracy. To evaluate the precision and reliability of our analyses, analytical uncertainties for both MeHg and THg analyses were obtained by instrument blanks during each operation, triplicated analyses for each sample, and operation/spike recoveries with standards for each batch (see Supplementary Texts S1 and S2).

## Supplementary Materials

### This PDF file includes:

Supplementary Text Figs. S1 to S9 Tables S1 to S8 References
