# Supplementary material for: Elevated methylmercury in Arctic rain and aerosol linked to air-sea exchange of dimethylmercury
Source: Sci Adv. 2025 Mar 19;11(12):eadr3805. doi: 10.1126/sciadv.adr3805 (PMC11922009; doi:10.1126/sciadv.adr3805)
Supplement: Supplementary file 1 — Supplementary Text Figs. S1 to S9 Tables S1 to S8 References [file sciadv.adr3805_sm.pdf]

Science Advances
AAAS

Supplementary Materials for

Elevated methylmercury in Arctic rain and aerosol linked to air-sea exchange of dimethylmercury

Yipeng He *et al.*

Corresponding author: Yipeng He, hyp@uconn.edu; Robert P. Mason, robert.mason@uconn.edu

Sci. Adv. **11**, eadr3805 (2025)
DOI: 10.1126/sciadv.adr3805

This PDF file includes:

Supplementary Text
Figs. S1 to S9
Tables S1 to S8
References

Supplementary Text

Supplementary Text S1. Tekran speciation system measurement QA/QC

The Tekran speciation system was positioned on the front rail of the 03 deck of the ship at a height of approximately 10 m above sea level, alongside the high-volume aerosol sampler (Supplementary Fig. S1). Before embarking on our research cruise, the whole system was leak and zero-air tested using the pump module and calibrated externally using a Tekran 2505 Hg source at the dock. Also, automatic calibration and manual calibration were performed using the internal permeation source (25 hr) and the injection Hg source (biweekly), respectively, and the recovery was detected with an average of 98% ± 5%.

As we ventured into the challenging environmental conditions of the north section of the Bering Sea and Bering Straits, we remained acutely aware of the potential impact of windy weather and rough seas on our equipment. Additional care was taken to keep sea spray out of the Tekran 1130 and 1135 cases. The shelters for all side vents were extended to 20-30 cm with hard-plastic board to prevent the sea spray from getting inside the instrument. Additionally, we covered the bottom vents on both instrument cases with duct tape to minimize variations within the cases.

The inlet, including an upgraded heated impactor to better remove the coarse particle (> 2.5 μm), was located at a vertical height of 40 cm from the deck's floor on the steel frame. To maintain a stable temperature and prevent potential Hg absorption and desorption at tubing walls due to temperature fluctuations, we ensured that the heated sample lines remained fully temperature controlled. Both units inside the system were equipped with heaters to prevent moisture or ice formation.

The detection limits (DL) for the Tekran speciation system were estimated at 0.02 ng m^{-3}, 1.0 pg m^{-3} and 1.0 pg m^{-3} for Hg^0, RGHg and Hg^P, respectively(*49, 69*). During our cruise, the blanks for Hg^P operations typically remained below 0.1 pg each cycle, effectively lowering the field detection limits for Hg^P to < 1 pg m^{-3}. Soda lime trap, in-line filters, a KCl-coated denuder and a particulate glass trap in the Tekran speciation system were installed at port of Dutch Harbor at the beginning of the cruise and were replaced in the Chukchi Sea at the middle of the cruise.

This study measured the Hg species on shipboard, unlike other studies on the land, which means the potential contamination from boat itself needs to be considered. To address this concern, we implemented a rigorous data discrimination process. We excluded data collected during two specific conditions: 1) when the ship was stationary at vertical profile stations along the transect, and 2) when the wind direction deviated more than ± 60° relative to the ship's bow. Adhering to the same operational criteria as the high-volume aerosol sampler, we effectively eliminated potential contamination originating from the ship's exhaust. Our post-cruise data validation procedures followed the protocol established by the Atmospheric Mercury Network (AMNet; https://nadp.slh.wisc.edu/).

Supplementary Text S2. Aerosol filter measurement QA/QC

The high-volume aerosol sampler was deployed on the front rail of the 03 deck of the ship at a height of approximately 10 m above sea level. This positioning, adjacent to the Tekran speciation system (see Supplementary Fig. S1), was pivotal for enabling direct comparison of particulate THg concentrations.

To minimize contamination risks, we adopted a meticulous process for handling aerosol filters. Aerosol filters were loaded into and unloaded from the Teflon filter pack in a High Efficiency Particulate Air (HEPA) filter blower, housed within a specially constructed plastic bubble clean space in the ship's laboratory to prevent any potential contamination from the inside

air. In addition, to prevent contamination from the ship's exhaust for the high-volume aerosol sampler, the pump was controlled by an automated sector-control system, using an anemometer interfaced with a Campbell Scientific CR800 datalogger, with the cycling between on and off being determined by the wind speed/direction relative to the ship speed/direction. This setup ensured that the sampler's pump was operational only when the relative wind direction was within ±60° of the ship's bow and the speed exceeded 0.5 m/s for a minimum of five continuous minutes. Such conditions, as suggested by Marsay et al. (2018)(*75*), are ideal for avoiding the ship's stack exhaust.

In addition, deployment blanks of the filter pack were collected during the cruise by loading but not deploying the filter packs and they were stored in the same manner as sample filters. For the aerosol sampler, eight filter packs were mounted on the tray and deployed each time. Therefore, the combination of three aerosol filters for each analysis was designed to minimize the uncertainty. However, due to the strong wind and rough sea, there is the possibility for capturing sea spray particles during some filter pack deployments. The average volume processed per filter is 379 m^3 and each deployment was about 2 to 3 days to obtain a signal above the detection limit. Detailed information for each deployment of the aerosol filters has been summarized in Supplementary Table S4.

All filters were stored frozen in polystyrene petri dishes which were double-bagged and transported to University of Connecticut, Avery Point campus for the analysis of MeHg and THg concentrations after the cruise. The digested aerosol filters were analyzed by the Tekran 2700 and Tekran 2600 for the MeHg and THg concentration, respectively. And the precision and recovery were determined by analyzing MeHg standards and THg standards, respectively. The mean recovery was 103 ± 7% for all MeHg standards, and 98 ± 9% for all THg standards.

The aerosol filters were made of glass fiber (GFF) which have been prebaked at 450 °C for 12 hr and stored wrapped in foil to prevent any Hg contamination during handling. The deployment blanks were determined for MeHg and THg, with < DL and 11 ± 5 pg each filter, respectively. Throughout the cruise, the variation of our blank concentrations was consistent. Also, the deployment blanks were in the same range as that of unused GFF filters (< 5% difference).

Supplementary Text S3. Air-sea exchange calculation of MeHg

Based on the high-resolution data of dissolved DMHg in surface waters, the DMHg evasion flux can be calculated based on its Henry's Law constant and a quadratic function of wind speed of the mass transfer coefficient for DMHg(*76*). The mass transfer coefficient is both temperature and wind speed dependent, and again the detailed calculation of their values for DMHg can be found in Supplementary Table S1. Wind speed data, sourced at a 10Hz frequency from the ship's anemometer and averaged to each minute, complemented the minute-averaged underway temperature data from the ship's sensors.

For parts of our cruise with the sea-ice covered in the Chukchi Sea, we still use the wind speed to estimate the flux, however, these calculations may not precisely represent the actual flux and should be considered an upper limit. Nevertheless, given the low dissolved DMHg concentration while the ship was in the sea-ice, we assume this calculation offers a reasonable representation for the flux balance calculation.

Dry deposition is calculated based on the aerosol-bound MeHg by the equation as

$$F_{DryDep,MeHg} = V_b \times C_{aerosol,MeHg}$$

where V_b denotes the corresponding aerosol dry deposition rate in the atmosphere. The dry deposition rate was estimated based on the aerosol size spectrum as the aerosol diameter is known

to be an important factor to determine its dry deposition rate. The aerosol size in this study was only categorized into two size groups – less than 2.5 μm and larger than 2.5 μm, which were quantified by the Tekran speciation system and the high-volume aerosol sampler. Assuming a uniform distribution of THg across these size groups, we derived the size distribution ratio from THg ratios obtained from these two measurements. This ratio was then applied to calculate the dry deposition rate, following the method outlined by Zhang and He (2014)(*77*).

Wet deposition is estimated based on the MeHg concentration in rainwater, which can be calculated by the equation:

$$F_{WetDep,MeHg} = R_{precipitation} \times C_{aerosol,MeHg}$$

where $R_{precipitation}$ represents the corresponding precipitation rate in the Bering Sea and Chukchi Sea during the cruise period. The precipitation rate was sourced from the ERA5 reanalysis dataset on single levels from 1940 to present (https://cds.climate.copernicus.eu), which integrates model data with global observations(*56*). Here, we extracted the precipitation data from 2012 to 2022 focusing on the spring season (April – June) to coincide with our Arctic cruise timeline, and averaged over the Aleutian Islands in the coastal Alaska region, south Bering Sea (55°N – 60°N), north Bering Sea (60°N – 65°N) and Chukchi Sea. The summarized fluxes of DMHg evasion and atmospheric MeHg deposition are detailed in Supplementary Table S6.

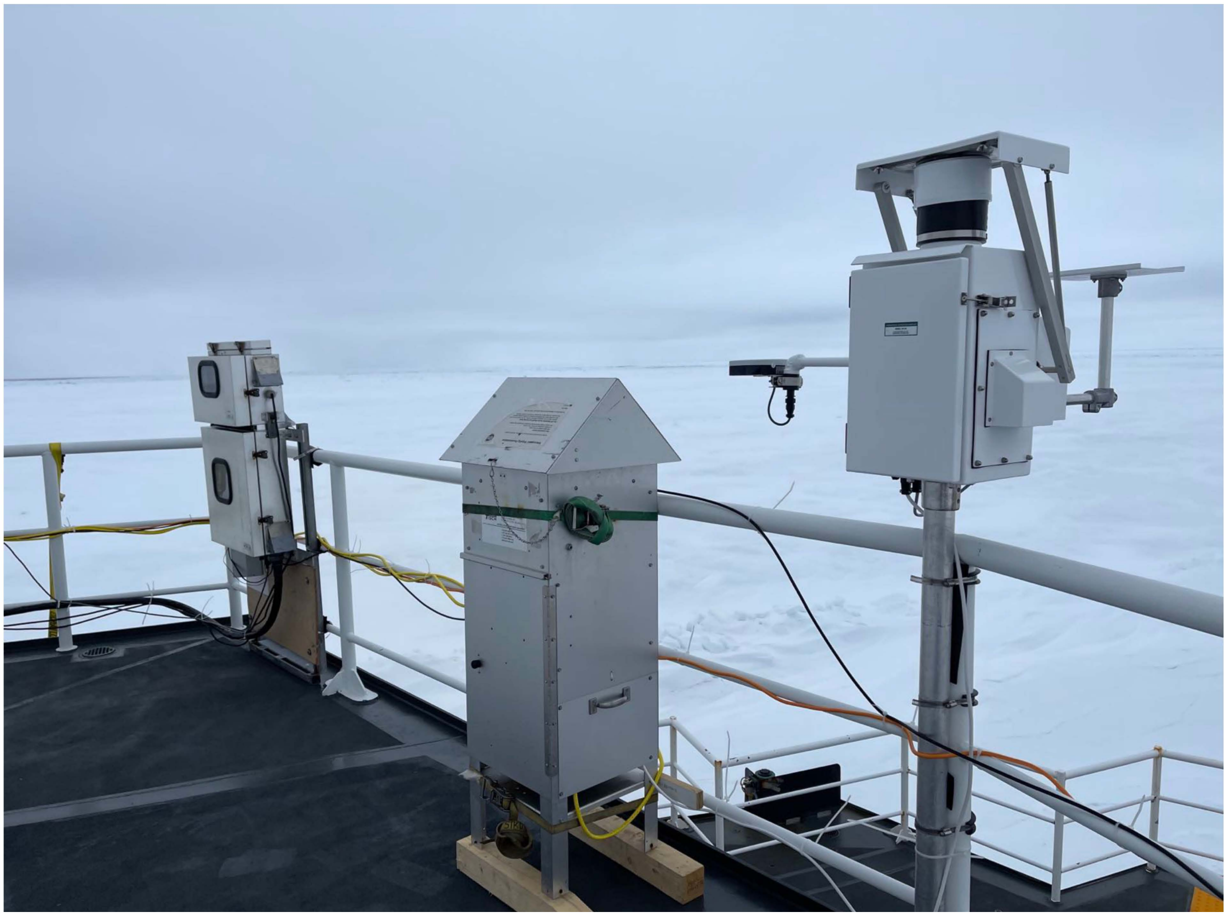

Supplementary Figure S1

Fig. S1.

A photo of the atmospheric apparatus on the front rail of the 03 deck of the ship. From left to right, they are the Tekran speciation system, high-volume aerosol sampler and N-CON rain sampler. Photo is taken by Dr. Yipeng He from University of Connecticut.

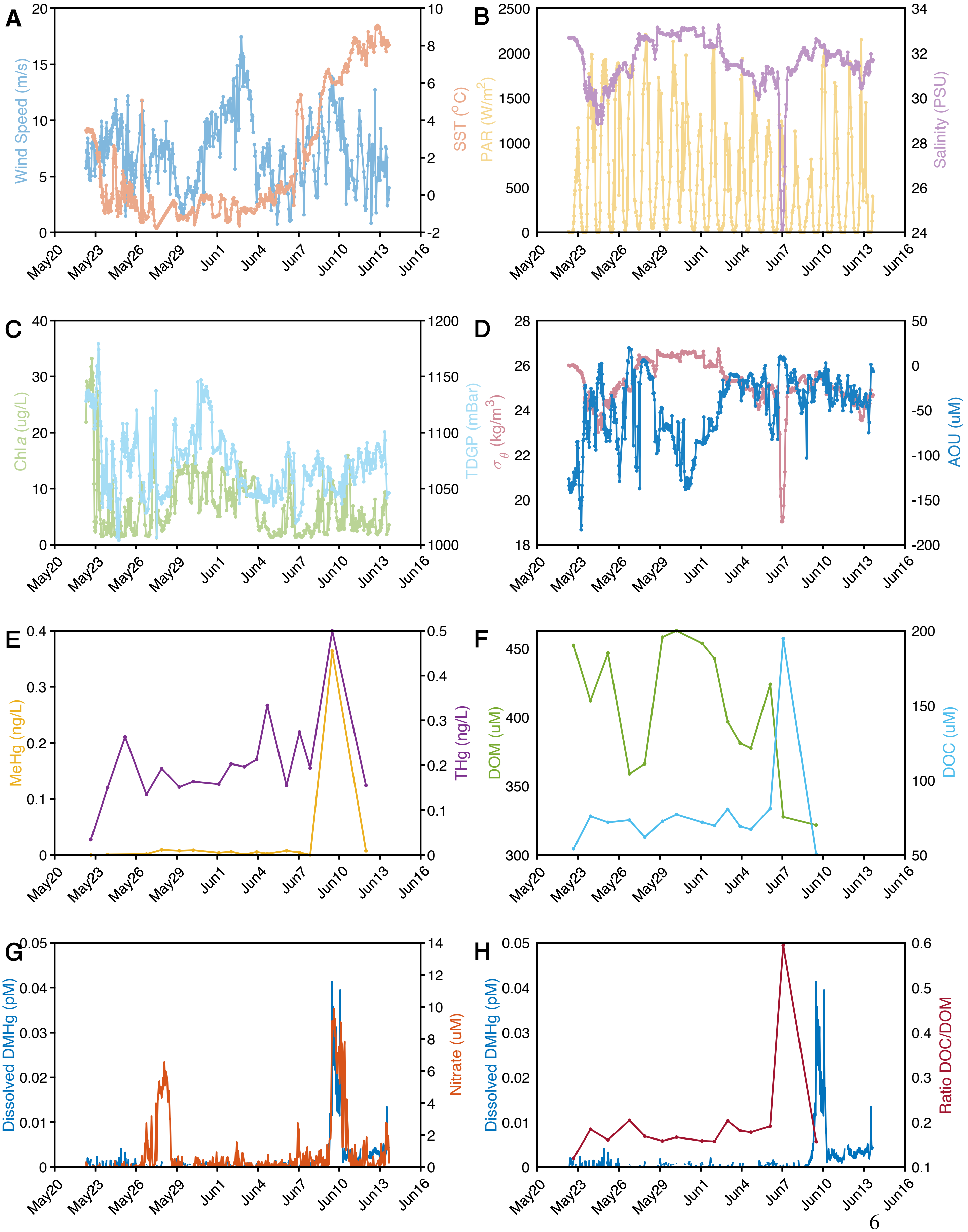

Supplementary Figure S2

Fig. S2.

Time series data of water mass properties and environmental parameters associated with dissolved Hg species along the cruise. (**A**) Wind speed (light blue line; left axis) in atmosphere and sea surface temperature (SST; light orange line; right axis) in surface seawater. (**B**) Photosynthetically active radiation (PAR; light yellow line; left axis) in atmosphere and salinity (light purple line; right axis) in surface seawater. (**C**) Chlorophyll-*a* (Chl*a*; light green line; left axis) and total dissolved gas pressure (TDGP; light blue line; right axis) in surface. (**D**) Potential density (Sigma; light red line; left axis) and apparent oxygen utilization (AOU; light blue line; right axis) in surface seawater. (**E**) Methylated mercury (MeHg; yellow line; left axis) and total mercury (THg; purple line; right axis) in surface seawater. (**F**) Dissolved organic matter (DOM; green line; left axis) and dissolved organic carbon (DOC; light blue line; right axis) in surface seawater. (**G**) Dissolved dimethylmercury (DMHg; blue line; left axis) and nitrate (orange line; right axis) in surface seawater. (**H**) Dissolved dimethylmercury (DMHg; blue line; left axis) and radios of DOC and DOM (red line; right axis) in surface seawater. The continuous underway monitoring data for environmental parameters were obtained from R/V *Sikuliaq* using ship-mounted instruments (details available at the website, https://www.sikuliaq.alaska.edu/ops/index). Additionally, the analysis data for dissolved organic carbon (DOC) and dissolved organic matter (DOM) are derived from Barret et al., 2023 (*78*).

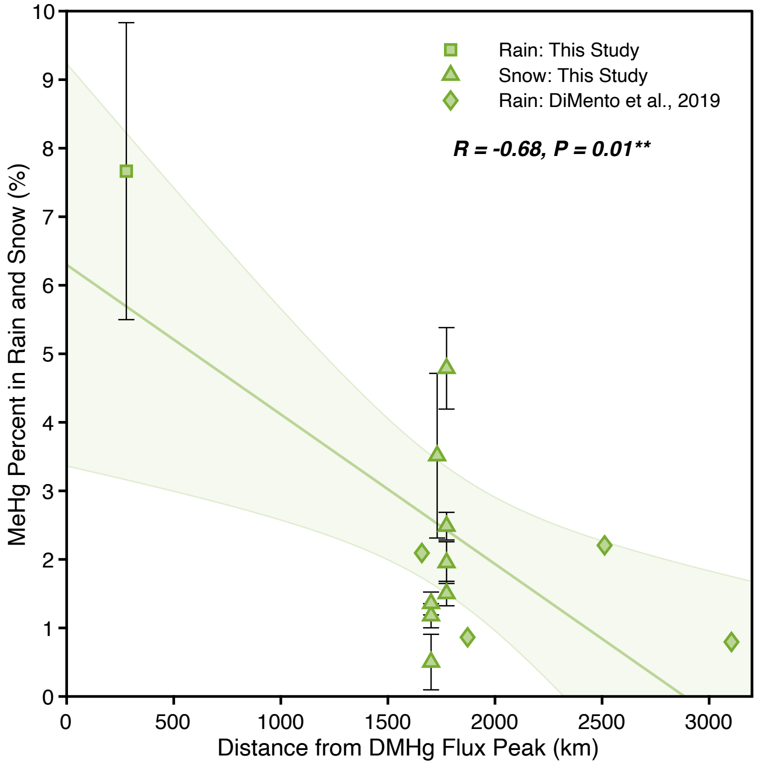

Supplementary Figure S3

Fig. S3.

The linear correlation between ratios of MeHg:THg in rain and snow and the distance from the DMHg flux peak. The linear correlation between measured ratios of MeHg:THg in rain (green square) and snow (green triangles) from this study and the U.S. Arctic GEOTRACES cruise(*27*) (green diamonds; Supplementary Table S5) and the distance from the DMHg flux peak. Robustness is denoted as *Pearson's R* and significance level is denoted as *** ($p < 0.001$), ** ($p < 0.01$), and * ($p < 0.05$).

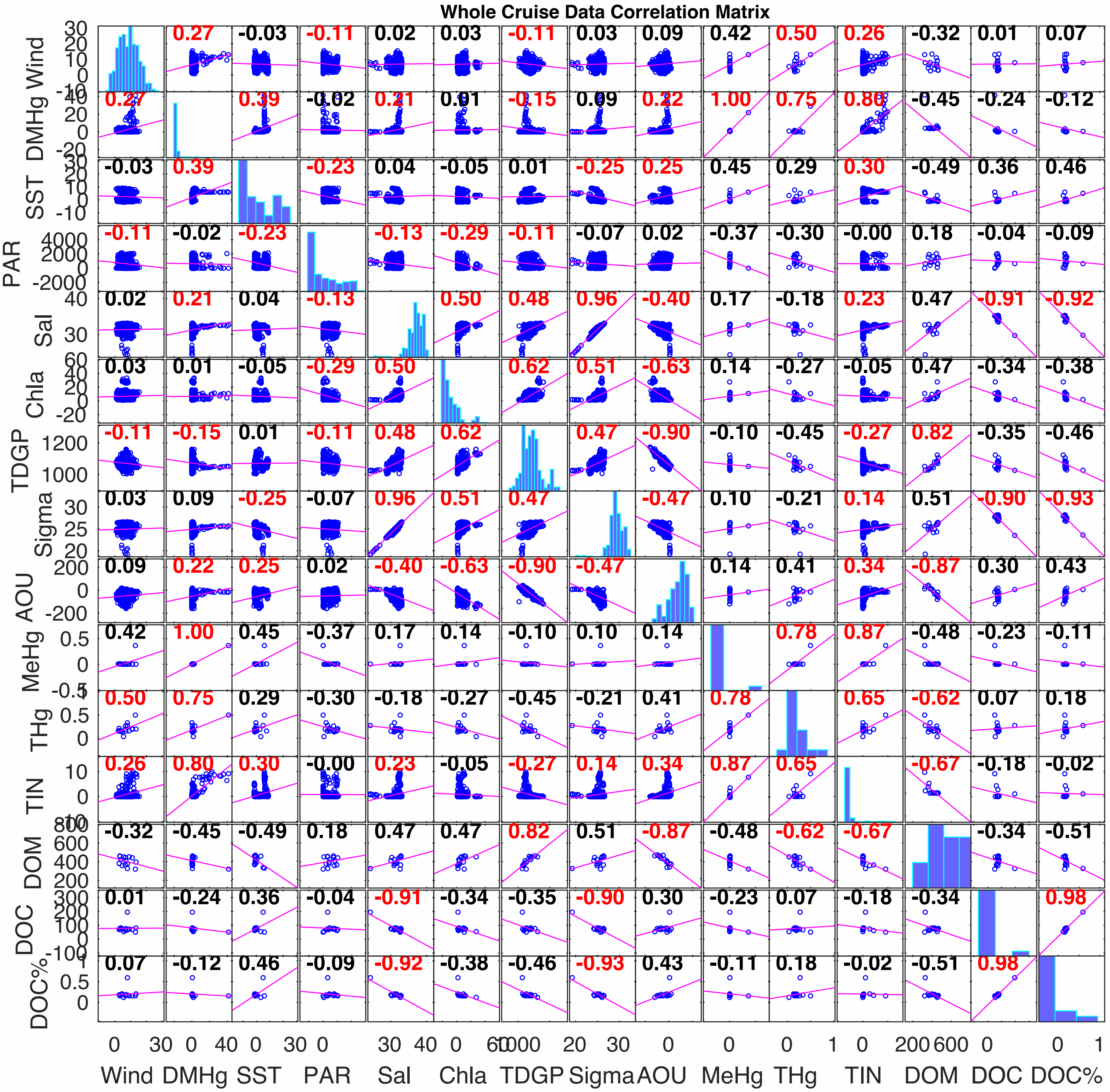

Supplementary Figure S4

Fig. S4.

A comprehensive correlation matrix between all pairs of underway measurements throughout the entire cruise. A comprehensive correlation matrix, featuring linear correlation assessments and bar distribution plots, for dissolved DMHg in conjunction with underway data of surface ocean and atmospheric conditions throughout the entire cruise. Numbers in red indicate a significant correlation between two parameters (t-test, $p < 0.05$*), while numbers in black denote an insignificant correlation (t-test, $p > 0.05$).

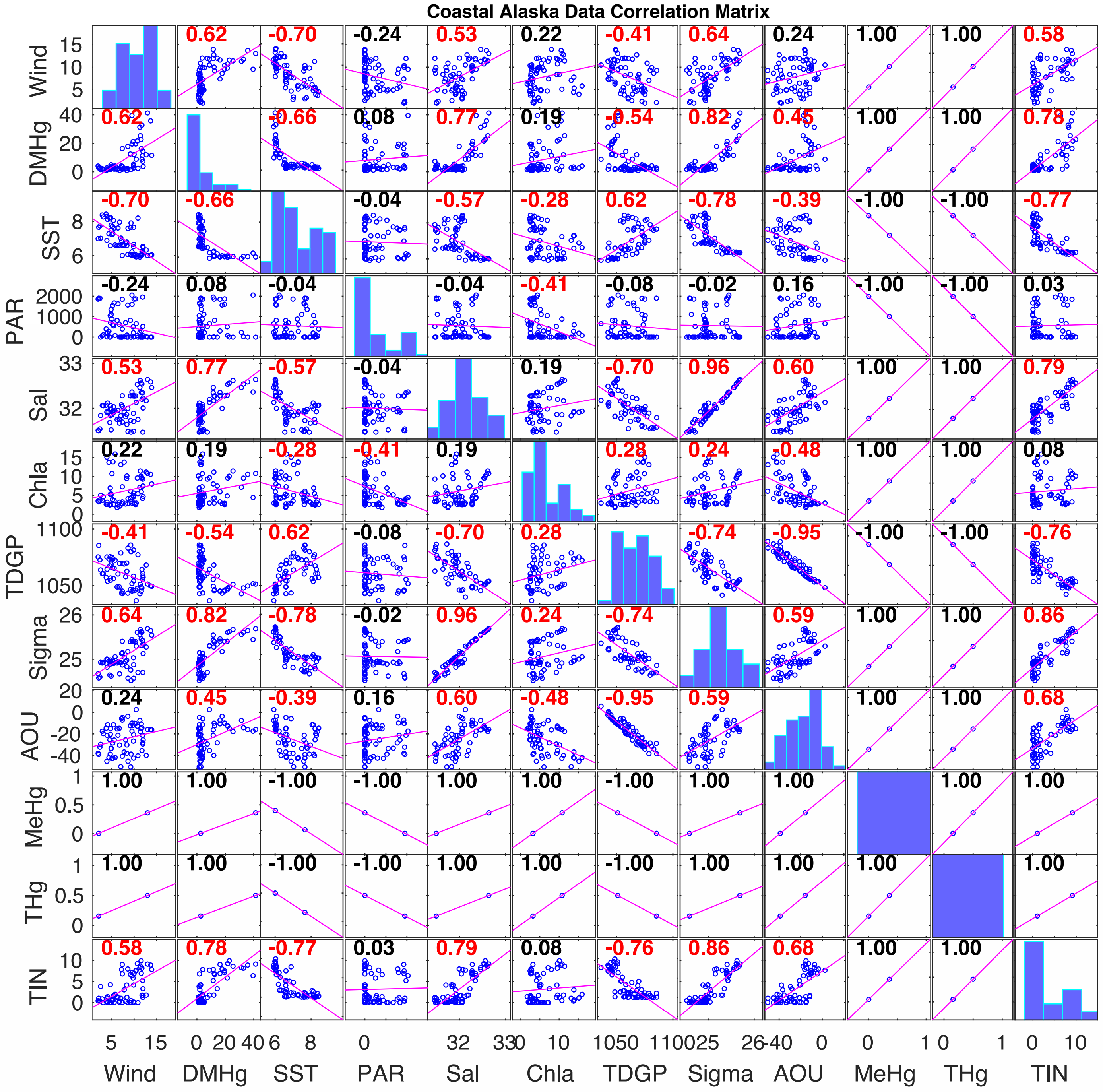

Supplementary Figure S5

Fig. S5.

A comprehensive correlation matrix between all pairs of underway measurements in the coastal Alaska region. A comprehensive correlation matrix, featuring linear correlation assessments and bar distribution plots, for dissolved DMHg in conjunction with underway data of surface ocean and atmospheric conditions in the coastal Alaska region. Numbers in red indicate a significant correlation between two parameters (t-test, $p < 0.05$*), while numbers in black denote an insignificant correlation (t-test, $p > 0.05$).

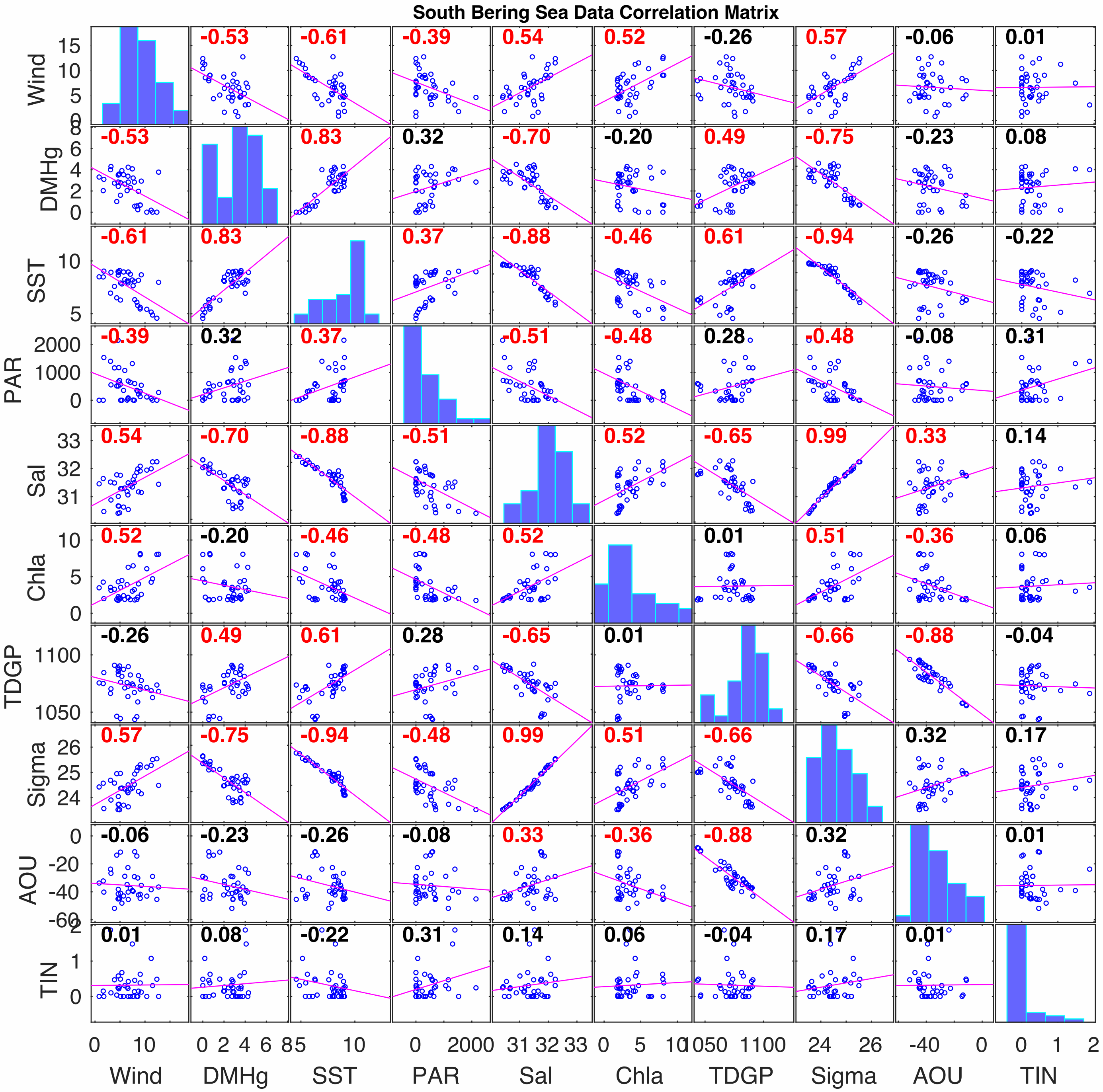

Supplementary Figure S6

Fig. S6.

A comprehensive correlation matrix between all pairs of underway measurements in the south Bering Sea. A comprehensive correlation matrix, featuring linear correlation assessments and bar distribution plots, for dissolved DMHg in conjunction with underway data of surface ocean and atmospheric conditions in the south Bering Sea. Numbers in red indicate a significant correlation between two parameters (t-test, $p < 0.05$*), while numbers in black denote an insignificant correlation (t-test, $p > 0.05$).

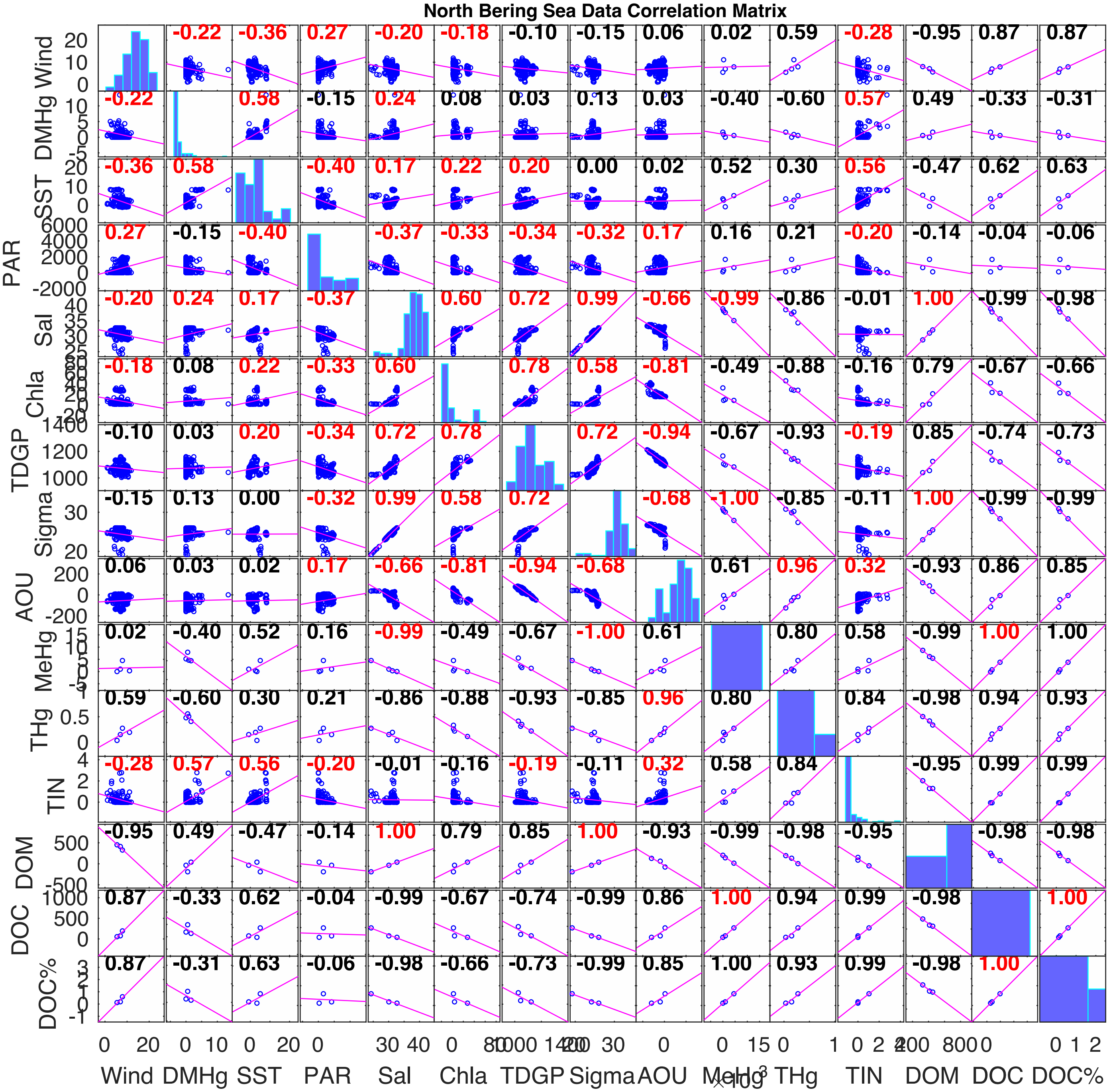

Supplementary Figure S7

Fig. S7.

A comprehensive correlation matrix between all pairs of underway measurements in the north Bering Sea. A comprehensive correlation matrix, featuring linear correlation assessments and bar distribution plots, for dissolved DMHg in conjunction with underway data of surface ocean and atmospheric conditions in the north Bering Sea. Numbers in red indicate a significant correlation between two parameters (t-test, $p < 0.05$*), while numbers in black denote an insignificant correlation (t-test, $p > 0.05$).

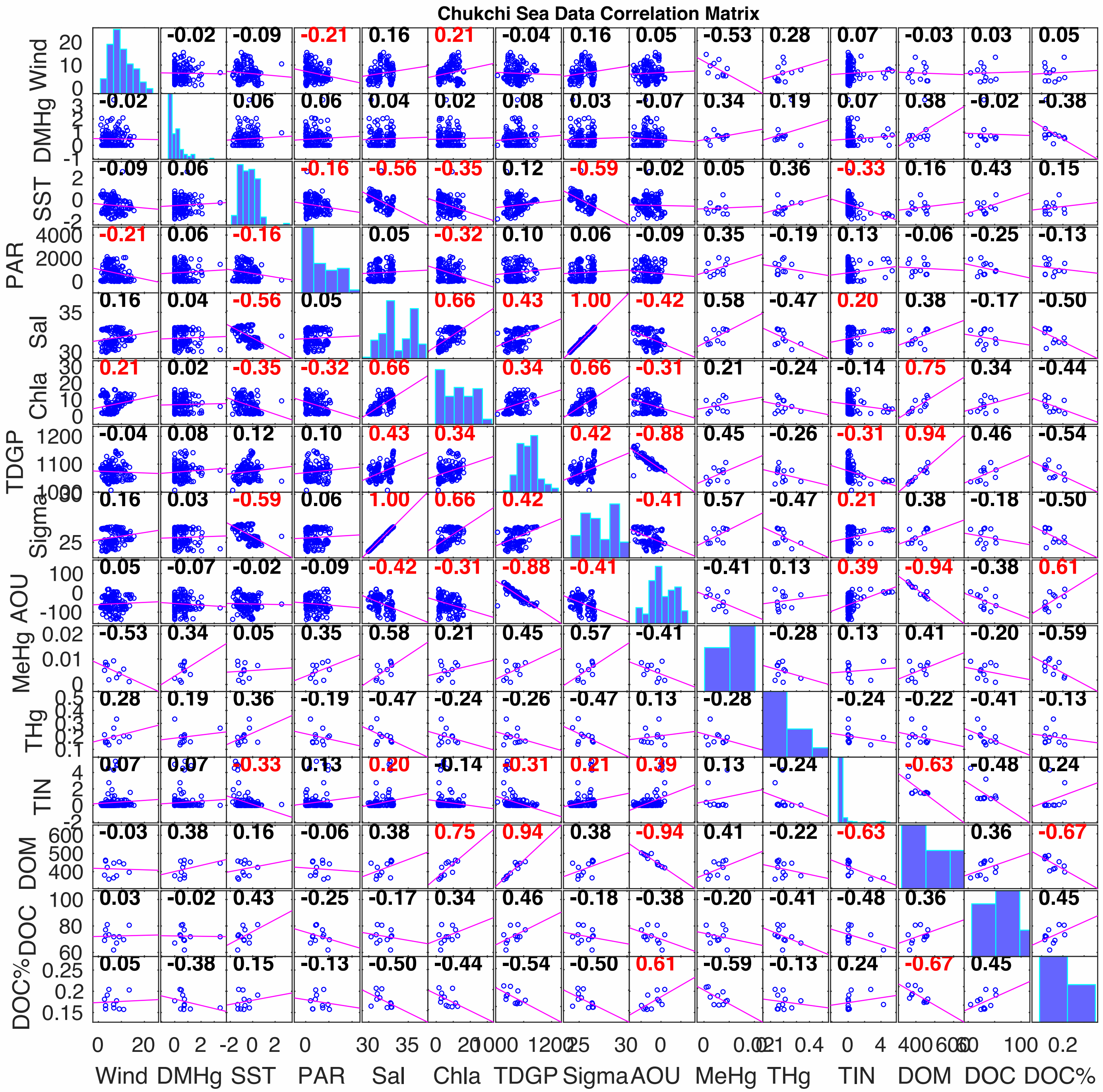

Supplementary Figure S8

Fig. S8.

A comprehensive correlation matrix between all pairs of underway measurements in the Chukchi Sea. A comprehensive correlation matrix, featuring linear correlation assessments and bar distribution plots, for dissolved DMHg in conjunction with underway data of surface ocean and atmospheric conditions in the Chukchi Sea. Numbers in red indicate a significant correlation between two parameters (t-test, $p < 0.05$*), while numbers in black denote an insignificant correlation (t-test, $p > 0.05$).

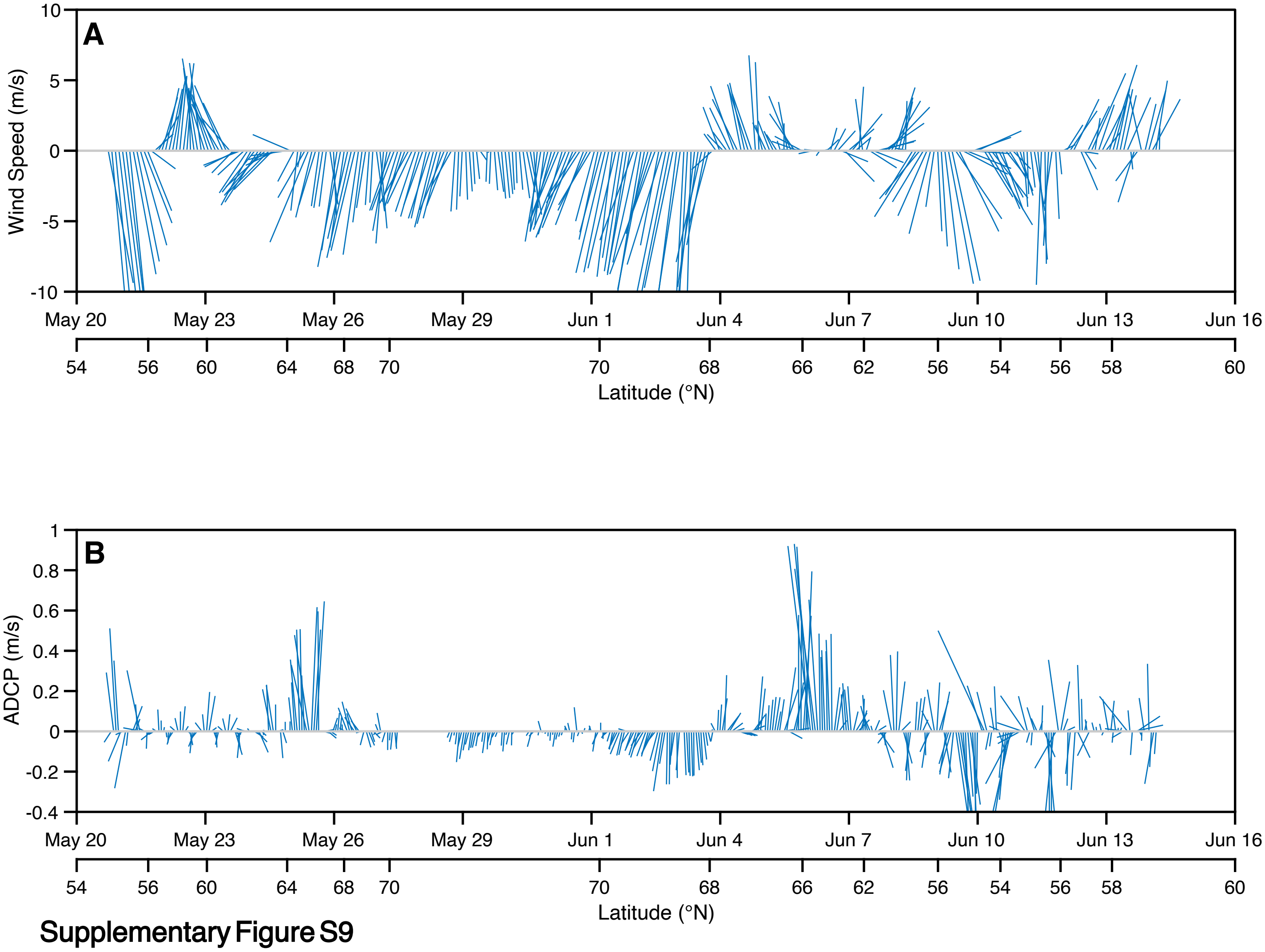

Fig. S9.

Wind speed in atmosphere and ADCP data from the surface ocean. Wind data (**A**) at 10 m height and ADCP data (**B**) from the surface ocean (< 25 m) along the ship track. The direction and magnitude are shown as the blue vector's direction and length.

Table S1.

Parameters and equations used for calculating the air-sea exchange flux of dimethylmercury (DMHg). This table summarizes the parameters, descriptions, equations, and references essential for determining the flux of DMHg across the air-sea interface. The references included in the table correspond to literature sources for the equations and parameters used in the calculations.

Parameter	Description	Equation	Ref.
F_{DMHg} (ng m^{-2} hr^{-1})	DMHg air-sea exchange flux	$k_{w,DMHg}\left(DMHg_{diss} - \frac{C_{air,DMHg}}{H_{DMHg}}\right)$	(*30*)
$k_{w,DMHg}$ (m hr^{-1})	Water side mass transfer coefficient for DMHg	$A \times \frac{u_{10}^2}{\sqrt{Sc_{DMHg}/Sc_{CO_2}}}$	(*76*)
H_{DMHg} (unitless)	Henry's law constant for DMHg	$\ln H_{DMHg} = -\frac{2512.43}{T} + 7.27$	(*79*)
T (K)	Temperature in Kelvin		
T' (°C)	Temperature in Celsius		
$DMHg_{diss}$ (ng m^{-3})	Dissolved DMHg concentration in seawater		
$C_{air,DMHg}$ (ng m^{-3})	DMHg concentration in air		
A (unitless)	Constant based on the Weibull distribution of wind speeds over oceans	0.25	(*80*)
u_{10}^2 (m s^{-1})	Wind speed normalized to 10 m above sea surface		(*81*)
Sc_{CO_2} (unitless)	Schmidt number for CO_2	$0.11T'^2 - 6.16T' + 644.7$	(*82*)
Sc_{DMHg} (unitless)	Schmidt number for DMHg	ν/D_{DMHg}	(*82*)
ν (cm^2 s^{-1})	Kinematic viscosity	$0.017e^{-0.025T'}$	(*82*)
D_{DMHg} (cm^2 s^{-1})	Diffusivity for DMHg	$\frac{7.4 \times 10^{-10} T \sqrt{\phi_w M_w}}{N V_{B,DMHg}^{0.6}}$	(*83*)
ϕ_w (unitless)	The effective molecular weight of the solvent with respect to the diffusion process	2.26	(*84*)
M_w (g mol^{-1})	Molecular weight of water	18.0	(*84*)
N (cP)	Viscosity of water	$\log N = \frac{1301}{998.333+8.1855(T'-20)+0.00585(T'-20)^2} - 3.30233$	(*85*)
$V_{B,DMHg}$ (cm^3 mol^{-1})	Molal volume of DMHg at its normal boiling temperature	72.11	(*86*)

Table S2.

Dissolved DMHg and its flux measured by the DAA during the Arctic cruise. Note: a) Date and Time are recorded in UTC time zone; b) Latitude in North and South denotes as '+' and '-'; c) Longitude in East and West denotes as '+' and '-'.

Date[a]	Time[a]	Lat[b]	Lon[c]	Wind Speed u10 (m/s)	Seawater Temp (°C)	Diss DMHg (pmol/m^3)	Flux DMHg (pmol/m^2/h)
21-05-22	07:44:54	58.23	-172.17	5.75	3.47		
21-05-22	08:14:54	58.31	-172.25	6.34	3.40		
21-05-22	08:41:41	58.38	-172.33	7.33	3.39	1.82E-05	1.22E-06
21-05-22	09:11:41	58.45	-172.41	7.13	3.44	1.14E+00	7.29E-02
21-05-22	09:41:41	58.53	-172.49	5.93	3.47	8.88E-01	3.92E-02
21-05-22	10:11:41	58.61	-172.58	8.06	3.50	2.05E+00	1.67E-01
21-05-22	10:41:41	58.69	-172.66	8.42	3.56	6.58E-01	5.87E-02
21-05-22	11:11:41	58.77	-172.75	7.96	3.52	1.79E+00	1.42E-01
21-05-22	11:41:41	58.84	-172.83	7.05	3.54	1.88E+00	1.17E-01
21-05-22	12:11:41	58.92	-172.92	6.29	3.50	1.40E+00	6.95E-02
21-05-22	12:41:41	59.00	-173.00	6.63	3.43	1.14E+00	6.29E-02
21-05-22	13:11:41	59.00	-173.00	5.20	3.44	1.23E+00	4.16E-02
21-05-22	13:41:41	59.00	-173.00	4.82	3.49	1.11E+00	3.22E-02
21-05-22	14:11:41	59.00	-173.00	4.70	3.48	1.63E-04	4.52E-06
21-05-22	14:41:41	59.00	-173.00	5.64	3.47	8.67E-01	3.46E-02
21-05-22	15:11:41	59.00	-173.00	4.65	3.48	6.69E-01	1.82E-02
21-05-22	15:41:41	59.00	-173.00	4.65	3.47	9.52E-01	2.58E-02
21-05-22	16:11:41	59.00	-173.00	5.45	3.37	1.62E+00	6.03E-02
21-05-22	16:41:41	59.00	-173.00	5.06	3.44	1.52E+00	4.88E-02
21-05-22	17:11:41	59.00	-173.00	5.06	3.45		
21-05-22	17:41:41	59.01	-172.99	7.52	3.46	2.46E+00	1.75E-01
21-05-22	18:11:41	59.08	-172.92	7.93	3.49		
21-05-22	18:41:41	59.15	-172.85	8.01	3.40		
21-05-22	19:11:41	59.23	-172.78	8.07	3.36		
21-05-22	19:41:41	59.30	-172.71	8.06	3.14	9.58E-04	7.75E-05
21-05-22	20:11:41	59.37	-172.63	8.08	3.18	6.74E-01	5.48E-02
21-05-22	20:41:41	59.44	-172.56	7.91	3.02		
21-05-22	21:11:41	59.52	-172.49	7.59	3.22	4.67E-04	3.35E-05
21-05-22	21:41:41	59.59	-172.42	7.63	3.13		
21-05-22	22:11:41	59.67	-172.34	5.53	3.22		
21-05-22	22:41:41	59.74	-172.26	5.68	3.04	8.59E-04	3.43E-05

21-05-22	23:11:41	59.82	-172.18	5.04	2.90		
21-05-22	23:41:41	59.89	-172.10	6.42	2.90		
21-05-23	00:11:41	59.97	-172.03	6.19	2.84		
21-05-23	00:41:41	60.00	-172.00	6.11	2.80		
21-05-23	01:11:41	60.05	-171.93	7.29	2.75		
21-05-23	01:41:41	60.12	-171.83	6.79	2.65		
21-05-23	02:11:41	60.19	-171.72	7.00	2.60		
21-05-23	02:41:41	60.26	-171.62	7.19	2.25	2.50E+00	1.57E-01
21-05-23	03:11:41	60.33	-171.52	7.03	2.11	1.48E-03	8.83E-05
21-05-23	03:41:41	60.39	-171.41	7.45	1.95		
21-05-23	04:11:41	60.46	-171.31	7.56	1.96		
21-05-23	04:41:41	60.53	-171.21	7.22	2.09		
21-05-23	05:11:41	60.59	-171.11	7.10	1.79		
21-05-23	05:41:41	60.66	-171.02	7.52	2.13		
21-05-23	06:11:41	60.73	-170.92	7.36	2.16	5.18E-05	3.41E-06
21-05-23	06:41:41	60.79	-170.82	7.46	2.27	1.02E-04	6.88E-06
21-05-23	07:11:41	60.85	-170.72	7.22	2.03	8.60E-01	5.42E-02
21-05-23	07:41:41	60.92	-170.62	8.05	1.52	1.31E-03	1.01E-04
21-05-23	08:11:41	60.98	-170.53	7.68	0.89	1.01E-03	6.99E-05
21-05-23	08:41:41	61.00	-170.50	7.19	0.46	1.34E-03	8.06E-05
21-05-23	09:11:41	61.05	-170.44	8.57	0.55		
21-05-23	09:41:41	61.12	-170.34	8.29	1.22	1.18E-03	9.60E-05
21-05-23	10:11:41	61.18	-170.24	8.44	1.34		
21-05-23	10:41:41	61.24	-170.13	9.92	1.21	2.62E-04	3.05E-05
21-05-23	11:11:41	61.30	-170.03	9.43	-0.02	6.83E-04	6.96E-05
21-05-23	11:41:41	61.33	-169.89	6.38	-0.13		
21-05-23	12:11:41	61.36	-169.75	6.80	0.33	1.47E+00	7.84E-02
21-05-23	12:41:41	61.43	-169.65	6.37	-0.41	1.81E-04	8.34E-06
21-05-23	13:11:41	61.45	-169.49	6.42	0.18	1.23E-03	5.85E-05
21-05-23	13:41:41	61.51	-169.37	8.13	-0.11	1.19E+00	8.96E-02
21-05-23	14:11:41	61.58	-169.30	7.28	-0.08	9.31E-04	5.63E-05
21-05-23	14:41:41	61.60	-169.17	6.25	-0.83	6.71E-04	2.94E-05
21-05-23	15:11:41	61.58	-169.01	5.12	-0.82		
21-05-23	15:41:41	61.57	-168.83	6.01	-0.54	1.67E+00	6.80E-02
21-05-23	16:11:41	61.59	-168.74	6.72	-0.94		
21-05-23	16:41:41	61.60	-168.69	7.38	-0.96		

21-05-23	17:11:41	61.64	-168.64	8.44	-0.55	1.70E-03	1.37E-04
21-05-23	17:41:41	61.68	-168.58	8.49	-0.65	3.25E-04	2.64E-05
21-05-23	18:11:41	61.71	-168.52	8.28	-0.61	1.10E-03	8.51E-05
21-05-23	18:41:41	61.73	-168.45	8.33	-0.64	6.55E-01	5.12E-02
21-05-23	19:11:41	61.80	-168.42	9.26	0.53	2.37E-04	2.36E-05
21-05-23	19:41:41	61.88	-168.45	9.77	0.06	1.58E+00	1.73E-01
21-05-23	20:11:41	61.94	-168.52	9.01	-0.34		
21-05-23	20:41:41	61.99	-168.54	8.49	-0.55	1.11E-03	9.03E-05
21-05-23	21:11:41	62.00	-168.55	6.66	-0.54	5.02E-04	2.51E-05
21-05-23	21:41:41	62.01	-168.56	6.94	-0.80	1.08E-03	5.85E-05
21-05-23	22:11:41	62.01	-168.57	7.27	-0.49	1.77E-03	1.06E-04
21-05-23	22:41:41	62.01	-168.59	7.23	-0.92	1.45E-03	8.49E-05
21-05-23	23:11:41	62.01	-168.60	7.49	-0.94	2.60E-03	1.63E-04
21-05-23	23:41:41	62.01	-168.62	7.95	-0.81		
21-05-24	00:11:41	62.01	-168.62	7.96	-0.52	9.56E-04	6.85E-05
21-05-24	00:41:41	62.01	-168.62	8.24	-0.40		
21-05-24	01:11:41	62.01	-168.62	8.79	-0.42		
21-05-24	01:41:41	62.01	-168.62	8.54	-0.30	6.00E-04	4.97E-05
21-05-24	02:11:41	62.03	-168.55	9.07	-0.34	6.31E-04	5.90E-05
21-05-24	02:41:41	62.06	-168.47	8.99	-0.35	1.47E+00	1.35E-01
21-05-24	03:11:41	62.09	-168.40	9.03	-0.79	2.72E-03	2.48E-04
21-05-24	03:41:41	62.11	-168.35	9.16	-0.84	1.52E+00	1.43E-01
21-05-24	04:11:41	62.14	-168.27	7.90	-0.22		
21-05-24	04:41:41	62.15	-168.21	8.53	-0.42		
21-05-24	05:11:41	62.20	-168.11	9.00	0.06	1.99E-03	1.85E-04
21-05-24	05:41:41	62.25	-168.01	9.31	-0.10	1.28E-03	1.27E-04
21-05-24	06:11:41	62.32	-167.96	9.55	0.08	1.23E-03	1.28E-04
21-05-24	06:41:41	62.40	-167.91	10.22	-0.03	3.16E-04	3.78E-05
21-05-24	07:11:41	62.47	-167.86	10.28	-0.19		
21-05-24	07:41:41	62.54	-167.82	10.38	1.94	3.35E-04	4.36E-05
21-05-24	08:11:41	62.62	-167.77	9.77	2.62	7.33E-04	8.59E-05
21-05-24	08:41:41	62.69	-167.73	10.13	2.64	7.23E-01	9.12E-02
21-05-24	09:11:41	62.77	-167.68	10.61	1.70	1.19E-03	1.60E-04
21-05-24	09:41:41	62.85	-167.63	10.40	2.38		
21-05-24	10:11:41	62.92	-167.58	9.74	2.48	6.38E-04	7.40E-05
21-05-24	10:41:41	62.99	-167.51	9.64	2.44		

21-05-24	11:11:41	63.00	-167.50	10.32	2.39	6.40E-04	8.31E-05
21-05-24	11:41:41	63.08	-167.51	11.05	2.45	1.52E-03	2.27E-04
21-05-24	12:11:41	63.16	-167.53	11.87	2.18	1.38E+00	2.36E-01
21-05-24	12:41:41	63.23	-167.52	10.27	1.49	2.06E-04	2.59E-05
21-05-24	13:11:41	63.27	-167.47	10.90	0.37		
21-05-24	13:41:41	63.30	-167.48	10.53	-0.81	3.67E-04	4.56E-05
21-05-24	14:11:41	63.35	-167.49	10.53	-0.56	8.66E-04	1.08E-04
21-05-24	14:41:41	63.40	-167.48	9.24	0.04		
21-05-24	15:11:41	63.43	-167.46	7.30	-0.47	2.73E-01	1.65E-02
21-05-24	15:41:41	63.46	-167.44	7.86	-1.14	5.27E-04	3.61E-05
21-05-24	16:11:41	63.51	-167.43	8.10	0.20	1.08E+00	8.13E-02
21-05-24	16:41:41	63.53	-167.37	7.93	-0.46	1.13E-03	8.00E-05
21-05-24	17:11:41	63.59	-167.33	8.56	0.00		
21-05-24	17:41:41	63.64	-167.28	7.51	-0.22	1.54E-04	9.85E-06
21-05-24	18:11:41	63.67	-167.20	9.17	-0.37	2.09E+00	1.99E-01
21-05-24	18:41:41	63.72	-167.18	9.13	-0.54	1.54E+00	1.45E-01
21-05-24	19:11:41	63.77	-167.15	8.34	-0.46		
21-05-24	19:41:41	63.82	-167.12	10.00	-0.72	1.47E+00	1.65E-01
21-05-24	20:11:41	63.88	-167.08	10.15	-0.20	1.50E-03	1.76E-04
21-05-24	20:41:41	63.92	-167.01	8.63	-0.93	2.02E-03	1.69E-04
21-05-24	21:11:41	63.96	-166.96	9.73	-0.98	1.89E-04	1.99E-05
21-05-24	21:41:41	64.00	-166.93	8.95	-0.59	4.33E+00	3.91E-01
21-05-24	22:11:41	64.05	-166.97	10.53	0.13		
21-05-24	22:41:41	64.13	-167.04	10.22	0.66		
21-05-24	23:11:41	64.22	-167.11	9.93	0.63	2.53E-04	2.91E-05
21-05-24	23:41:41	64.30	-167.18	9.81	0.60		
21-05-25	00:11:41	64.38	-167.25	9.33	0.71	1.98E-03	2.01E-04
21-05-25	00:41:41	64.47	-167.28	7.90	1.30	0.00E+00	0.00E+00
21-05-25	01:11:41	64.55	-167.29	5.25	1.10		
21-05-25	01:41:41	64.64	-167.33	5.29	0.05		
21-05-25	02:11:41	64.72	-167.39	2.98	-0.14		
21-05-25	02:41:41	64.80	-167.46	3.64	-0.19	5.69E-01	8.57E-03
21-05-25	03:11:41	64.89	-167.54	4.59	0.98	1.23E+00	3.06E-02
21-05-25	03:41:41	64.97	-167.64	6.02	0.26		
21-05-25	04:11:41	65.00	-167.68	6.54	-0.37		
21-05-25	04:41:41	65.01	-167.69	6.70	-0.29	3.45E+00	1.76E-01

21-05-25	05:11:41	65.01	-167.69	1.99	0.79	9.42E-04	4.37E-06
21-05-25	05:41:41	65.01	-167.69	1.48	0.32	2.04E+00	5.13E-03
21-05-25	06:11:41	65.01	-167.70	2.83	0.16	6.82E-04	6.29E-06
21-05-25	06:41:41	65.08	-167.66	7.35	0.37	5.45E-04	3.40E-05
21-05-25	07:11:41	65.16	-167.64	9.50	0.46		
21-05-25	07:41:41	65.24	-167.65	9.18	0.48		
21-05-25	08:11:41	65.33	-167.69	4.50	0.41	2.99E-01	7.01E-03
21-05-25	08:41:41	65.40	-167.80	8.64	0.25	1.21E+00	1.04E-01
21-05-25	09:11:41	65.46	-167.96	11.72	0.37		
21-05-25	09:41:41	65.50	-168.10	12.19	-0.54		
21-05-25	10:11:41	65.57	-168.25	11.39	-0.11	1.19E-03	1.77E-04
21-05-25	10:41:41	65.65	-168.32	9.03	-0.11	1.78E+00	1.66E-01
21-05-25	11:11:41	65.74	-168.29	6.74	-0.34		
21-05-25	11:41:41	65.82	-168.23	6.91	0.32	8.77E-01	4.84E-02
21-05-25	12:11:41	65.90	-168.18	5.93	0.68		
21-05-25	12:41:41	65.99	-168.13	5.61	-0.11	1.93E-04	6.94E-06
21-05-25	13:11:41	66.01	-168.10	6.79	0.21	1.23E-03	6.56E-05
21-05-25	13:41:41	66.09	-168.11	5.90	0.17	1.99E-03	7.98E-05
21-05-25	14:11:41	66.16	-168.19	5.84	-0.22	6.73E-04	2.62E-05
21-05-25	14:41:41	66.24	-168.29	4.68	-0.66		
21-05-25	15:11:41	66.32	-168.30	4.25	-0.64	1.61E-04	3.28E-06
21-05-25	15:41:41	66.39	-168.17	5.15	0.52		
21-05-25	16:11:41	66.42	-167.99	5.92	0.54	1.41E-03	5.74E-05
21-05-25	16:41:41	66.44	-167.80	7.15	0.41		
21-05-25	17:11:41	66.47	-167.67	5.13	0.21		
21-05-25	17:41:41	66.54	-167.60	5.17	-0.19	1.40E-03	4.27E-05
21-05-25	18:11:41	66.59	-167.51	5.21	-0.13	1.87E-03	5.80E-05
21-05-25	18:41:41	66.65	-167.47	5.95	-0.37	8.00E-01	3.21E-02
21-05-25	19:11:41	66.70	-167.46	6.16	-0.64	1.58E-03	6.76E-05
21-05-25	19:41:41	66.78	-167.43	4.35	-0.58	4.42E-04	9.46E-06
21-05-25	20:11:41	66.87	-167.41	3.34	-0.65	1.62E-05	2.05E-07
21-05-25	20:41:41	66.95	-167.41	4.03	-0.60	1.05E-03	1.93E-05
21-05-25	21:11:41	67.00	-167.42	4.49	-0.63	1.05E-03	2.40E-05
21-05-25	21:41:41	67.06	-167.42	4.82	-0.43	1.03E-03	2.72E-05
21-05-25	22:11:41	67.15	-167.42	5.09	-0.11	1.65E-03	4.89E-05
21-05-25	22:41:41	67.23	-167.37	5.23	-0.49	1.90E+00	5.88E-02

21-05-25	23:11:41	67.31	-167.39	5.57	-0.81	0.00E+00	0.00E+00
21-05-25	23:41:41	67.37	-167.50	4.92	-0.63	7.42E-01	2.03E-02
21-05-26	00:11:41	67.45	-167.60	6.44	-0.82	1.13E-03	5.26E-05
21-05-26	00:41:41	67.51	-167.73	6.30	-0.32	1.25E-03	5.61E-05
21-05-26	06:26:44	68.04	-168.41	8.86	-1.21		
21-05-26	07:26:44	68.11	-168.41	10.41	-1.39	1.52E-04	1.82E-05
21-05-26	08:26:44	68.14	-168.42	8.75	-1.44		
21-05-26	09:26:44	68.18	-168.52	10.49	2.58	9.45E-01	1.28E-01
21-05-26	10:26:44	68.25	-168.54	11.72	5.08		
21-05-26	11:26:44	68.35	-168.59	9.27			
21-05-26	12:26:44	68.46	-168.60	10.84	-1.45		
21-05-26	13:26:44	68.58	-168.38	9.52	-0.84		
21-05-26	14:26:44	68.69	-168.15	7.93	-0.97		
21-05-26	15:26:44	68.81	-167.90	5.13	-1.03		
21-05-26	16:26:44	68.89	-167.62	5.95	-1.74	2.58E-01	9.99E-03
21-05-26	17:26:44	68.99	-167.36	4.88	-1.70		
21-05-26	18:26:44	69.00	-167.34	4.56	-1.66	4.04E-01	9.23E-03
21-05-26	19:26:44	69.00	-167.33	4.18	-1.63	5.18E-01	9.94E-03
21-05-26	20:26:44	69.00	-167.32	4.20	-1.59		
21-05-26	21:26:44	69.00	-167.32	3.99	-1.55	6.39E-01	1.12E-02
21-05-26	22:26:44	69.04	-167.30	3.71	-1.52		
21-05-26	23:26:44	69.17	-167.10	4.85	-1.48		
21-05-27	00:26:44	69.27	-166.74	5.41	-1.44		
21-05-27	01:26:44	69.35	-166.35	6.69	-1.40		
21-05-27	02:26:44	69.47	-166.16	5.96	-1.37	2.02E+00	7.93E-02
21-05-27	03:26:44	69.53	-166.01	6.86	-1.33		
21-05-27	04:26:44	69.62	-165.80	7.00	-1.29		
21-05-27	05:26:44	69.77	-165.65	6.77	-1.26		
21-05-27	06:26:44	69.92	-165.53	9.15	-1.22		
21-05-27	07:26:44	70.04	-165.64	7.44	-1.18	3.77E-04	2.31E-05
21-05-27	08:26:44	70.04	-165.82	8.67	-1.15	1.63E-04	1.36E-05
21-05-27	09:26:44	70.04	-165.95	7.46	-1.11		
21-05-27	10:26:44	70.04	-165.97	7.00	-1.07	6.05E-04	3.31E-05
21-05-27	11:26:44	70.03	-166.01	8.28	-1.03		
21-05-27	12:26:44	70.03	-166.01	5.47	-1.00	2.70E-04	9.02E-06
21-05-27	13:26:44	70.03	-166.03	5.93	-0.96	3.88E-01	1.52E-02

21-05-27	14:26:44	70.03	-166.06	5.52	-0.92		
21-05-27	15:26:44	70.02	-166.07	4.40	-0.89	6.12E-01	1.32E-02
21-05-27	16:26:44	70.02	-166.08	6.80	-0.85		
21-05-27	17:26:44	70.02	-166.08	7.42	-0.81	7.84E-01	4.84E-02
21-05-27	18:26:44	70.02	-166.09	7.67	-0.78		
21-05-27	19:26:44	70.02	-166.10	7.18	-0.74		
21-05-27	20:26:44	70.02	-166.11	8.24	-0.70	3.12E-04	2.38E-05
21-05-27	21:26:44	70.02	-166.13	6.62	-0.66	7.34E-01	3.62E-02
21-05-27	22:26:44	70.01	-166.14	7.96	-0.63	1.25E+00	8.90E-02
21-05-27	23:26:44	70.01	-166.15	6.81			
21-05-28	00:26:44	70.01	-166.16	6.91			
21-05-28	01:26:44	70.01	-166.18	7.68			
21-05-28	02:26:44	70.00	-166.16	7.91			
21-05-28	03:26:44	69.99	-166.16	5.50			
21-05-28	04:26:44	69.98	-166.16	8.10			
21-05-28	05:26:44	69.96	-166.12	9.91			
21-05-28	06:26:44	69.96	-166.13	8.00			
21-05-28	07:26:44	69.95	-166.13	7.46			
21-05-28	08:26:44	69.95	-166.14	7.79			
21-05-28	09:26:44	69.94	-166.15	8.11			
21-05-28	10:26:44	69.94	-166.17	6.52			
21-05-28	11:26:44	69.94	-166.18	6.71			
21-05-28	12:26:44	69.91	-166.17	3.42			
21-05-28	13:26:44	69.90	-166.18	6.17			
21-05-28	14:26:44	69.88	-166.19	7.40			
21-05-28	15:26:44	69.83	-166.10	6.60			
21-05-28	16:26:44	69.86	-165.97	7.91			
21-05-28	17:26:44	69.96	-165.73	7.00			
21-05-28	18:26:44	70.07	-165.41	5.31			
21-05-28	19:26:44	70.17	-165.06	5.47	-0.60		
21-05-28	20:26:44	70.25	-164.67	5.82	-0.41	2.41E-01	9.23E-03
21-05-28	21:26:44	70.30	-164.24	4.87	-0.44	4.47E-01	1.20E-02
21-05-28	22:26:44	70.40	-163.95	3.93	-0.48		
21-05-28	23:26:44	70.47	-163.96	3.32	-0.97	6.27E-01	7.70E-03
21-05-29	00:26:44	70.47	-164.00	3.92	-1.13	1.82E+00	3.11E-02
21-05-29	01:26:44	70.46	-164.03	4.06	-1.27	4.45E-01	8.14E-03

21-05-29	02:26:44	70.46	-164.05	3.31	-1.32	5.75E-05	6.96E-07
21-05-29	03:26:44	70.46	-164.06	3.17	-1.36		
21-05-29	04:26:44	70.45	-164.07	3.14	-1.31	4.53E-01	4.93E-03
21-05-29	05:26:44	70.45	-164.08	2.25	-1.07	5.01E-01	2.82E-03
21-05-29	06:26:44	70.45	-164.09	2.62	-1.09		
21-05-29	07:26:44	70.45	-164.09	2.11	-1.12	8.59E-01	4.25E-03
21-05-29	08:26:44	70.45	-164.09	1.44	-1.18	2.45E-04	5.64E-07
21-05-29	09:26:44	70.44	-164.09	2.51	-1.22		
21-05-29	10:26:44	70.44	-164.10	1.73	-1.24	1.12E+00	3.71E-03
21-05-29	11:26:44	70.43	-164.10	1.54	-0.93	5.06E-01	1.33E-03
21-05-29	12:26:44	70.43	-164.10	1.58	-0.86		
21-05-29	13:26:44	70.43	-164.11	2.15	-0.79	3.32E-04	1.73E-06
21-05-29	14:26:44	70.42	-164.12	2.49	-0.73	2.79E-05	1.95E-07
21-05-29	15:26:44	70.42	-164.13	3.45	-0.66	8.53E-01	1.14E-02
21-05-29	16:26:44	70.42	-164.14	3.13	-0.60		
21-05-29	17:26:44	70.42	-164.15	3.42	-0.53		
21-05-29	18:26:44	70.42	-164.15	3.46	-0.46	2.33E-04	3.15E-06
21-05-29	19:26:44	70.42	-164.15	3.74	-0.40	6.16E-01	9.77E-03
21-05-29	20:26:44	70.42	-164.15	4.87	-0.33		
21-05-29	21:26:44	70.41	-164.15	3.85	-0.27	3.42E-01	5.76E-03
21-05-29	22:26:44	70.42	-164.15	4.00	-0.20		
21-05-29	23:26:44	70.41	-164.16	3.80	-0.94	4.60E-01	7.41E-03
21-05-30	00:26:44	70.41	-164.16	4.10	-1.01	7.05E-01	1.32E-02
21-05-30	01:26:44	70.40	-164.17	3.58	-0.86		
21-05-30	02:26:44	70.40	-164.18	3.54	-0.83		
21-05-30	03:26:44	70.39	-164.19	2.99	-0.75	8.21E-01	8.26E-03
21-05-30	04:26:44	70.39	-164.20	3.73	-0.66		
21-05-30	05:26:44	70.38	-164.20	3.11	-0.69	7.23E-01	7.87E-03
21-05-30	06:26:44	70.36	-164.20	1.87	-0.93	7.89E-01	3.08E-03
21-05-30	07:26:44	70.36	-164.20	3.28			
21-05-30	08:26:44	70.36	-164.21	4.05			
21-05-30	09:26:44	70.36	-164.22	4.58			
21-05-30	10:26:44	70.36	-164.22	4.58			
21-05-30	11:26:44	70.33	-164.24	3.57			
21-05-30	12:26:44	70.32	-164.26	3.93			
21-05-30	13:26:44	70.25	-164.27	5.54			

21-05-30	14:26:44	70.14	-164.55	6.25			
21-05-30	15:26:44	70.13	-165.00	4.62			
21-05-30	16:26:44	70.14	-165.05	5.28			
21-05-30	17:26:44	70.13	-165.06	7.35			
21-05-30	18:26:44	70.13	-165.08	6.98			
21-05-30	19:26:44	70.08	-164.97	7.59	-0.14	7.95E-01	5.23E-02
21-05-30	20:26:44	70.08	-164.92	7.62	-0.01	4.40E-01	2.92E-02
21-05-30	21:26:44	70.09	-164.73	6.32	-0.02		
21-05-30	22:26:44	70.10	-164.71	6.36	-0.14		
21-05-30	23:26:44	70.11	-164.78	2.57	-0.23		
21-05-31	00:26:44	70.11	-164.80	3.59	-0.33	7.84E-01	1.15E-02
21-05-31	01:26:44	70.11	-164.82	8.04	-0.22		
21-05-31	02:26:44	70.11	-164.85	9.84	-0.13	8.87E-01	9.81E-02
21-05-31	03:26:44	70.10	-164.84	8.25	-0.03	5.41E-01	4.21E-02
21-05-31	04:26:44	70.11	-164.85	7.52	0.01	1.39E+00	9.01E-02
21-05-31	05:26:44	70.11	-164.88	10.15	-0.11		
21-05-31	06:26:44	70.10	-164.91	8.00	-0.06	7.74E-04	5.67E-05
21-05-31	07:26:44	70.10	-164.94	8.87	-0.15	8.22E-01	7.38E-02
21-05-31	08:26:44	70.10	-164.96	9.84	-0.22		
21-05-31	09:26:44	70.10	-164.95	9.26	-0.19		
21-05-31	10:26:44	70.07	-164.96	7.71	-0.16		
21-05-31	11:26:44	70.07	-164.98	9.80	-0.04		
21-05-31	12:26:44	70.09	-165.00	10.58	-0.21		
21-05-31	13:26:44	70.10	-165.01	9.75	-0.15		
21-05-31	14:26:44	70.12	-165.27	10.74	-0.50	7.83E-03	1.02E-03
21-05-31	15:26:44	70.15	-165.28	8.61	-1.01	3.23E-01	2.67E-02
21-05-31	16:26:44	70.16	-165.30	7.66	-1.17	1.94E-04	1.26E-05
21-05-31	17:26:44	70.16	-165.31	8.47	-1.15		
21-05-31	18:26:44	70.16	-165.34	8.44	-1.14	1.61E+00	1.27E-01
21-05-31	19:26:44	70.16	-165.36	8.88	-1.15		
21-05-31	20:26:44	70.16	-165.38	10.54	-1.16		
21-05-31	21:26:44	70.15	-165.40	9.29	-1.15	2.57E-01	2.46E-02
21-05-31	22:26:44	70.15	-165.42	9.68	-1.14		
21-05-31	23:26:44	70.15	-165.44	10.27	-1.13	9.42E-01	1.11E-01
21-06-01	00:26:44	70.14	-165.46	8.70	-1.07	5.86E-02	4.94E-03
21-06-01	01:26:44	70.14	-165.48	8.25	-1.08		

21-06-01	02:26:44	70.14	-165.50	9.08	-1.06	1.19E+00	1.09E-01
21-06-01	03:26:44	70.11	-165.53	8.58	-1.01	3.76E-01	3.09E-02
21-06-01	04:26:44	70.00	-165.73	6.73	-0.37		
21-06-01	05:26:44	69.86	-166.03	7.02	-0.67		
21-06-01	06:26:44	69.78	-166.28	11.11	0.01		
21-06-01	07:26:44	69.75	-166.59	11.27	-0.14	6.01E-04	8.71E-05
21-06-01	08:26:44	69.75	-166.60	11.59	-0.15	4.10E-04	6.28E-05
21-06-01	09:26:44	69.75	-166.60	11.20	-0.24		
21-06-01	10:26:44	69.76	-166.61	12.12	-0.19	1.36E+00	2.28E-01
21-06-01	11:26:44	69.76	-166.62	10.54	-0.21		
21-06-01	12:26:44	69.76	-166.63	11.30	-0.24		
21-06-01	13:26:44	69.76	-166.69	11.31	-0.24		
21-06-01	14:26:44	69.78	-166.74	9.85	-1.36	4.24E-01	4.55E-02
21-06-01	15:26:44	69.74	-166.83	10.07	-0.74	1.63E-04	1.86E-05
21-06-01	16:26:44	69.81	-166.61	10.07	-0.85	3.60E-01	4.09E-02
21-06-01	17:26:44	69.81	-166.73	10.68	-1.30	2.08E+00	2.63E-01
21-06-01	18:26:44	69.81	-166.75	11.38	-1.46	8.50E-01	1.21E-01
21-06-01	19:26:44	69.80	-166.78	11.73	-1.18		
21-06-01	20:26:44	69.79	-166.82	10.82	-1.26		
21-06-01	21:26:44	69.78	-166.86	11.82	-1.39	2.18E-05	3.36E-06
21-06-01	22:26:44	69.77	-166.90	10.68	-1.34	6.99E-01	8.81E-02
21-06-01	23:26:44	69.76	-166.94	11.98	-1.30		
21-06-02	00:26:44	69.75	-166.98	11.00	-0.78	6.88E-01	9.35E-02
21-06-02	01:26:44	69.71	-167.01	9.92	-0.70		
21-06-02	02:26:44	69.70	-167.03	10.00	-0.50		
21-06-02	03:26:44	69.69	-167.01	10.44	-0.22		
21-06-02	04:26:44	69.70	-166.65	13.56	-0.23	4.60E-01	9.65E-02
21-06-02	05:26:44	69.73	-166.25	13.72	-0.14		
21-06-02	06:26:44	69.74	-165.83	13.06	-0.34		
21-06-02	07:26:44	69.75	-165.61	5.67	-0.43		
21-06-02	08:26:44	69.62	-165.97	8.74	-0.51		
21-06-02	09:26:44	69.48	-166.33	8.10	-0.44	6.01E-05	4.47E-06
21-06-02	10:26:44	69.42	-166.52	13.85	-0.43	6.62E-01	1.44E-01
21-06-02	11:26:44	69.51	-166.84	14.88	-0.37		
21-06-02	12:26:44	69.59	-167.20	12.51	-0.88		
21-06-02	13:26:44	69.49	-167.53	9.12	-1.53		

21-06-02	14:26:44	69.35	-167.76	9.25	-1.53	5.27E-04	4.96E-05
21-06-02	15:26:44	69.31	-167.83	12.74	-1.65		
21-06-02	16:26:44	69.31	-167.70	15.47	-1.01	6.56E-01	1.75E-01
21-06-02	17:26:44	69.16	-167.55	15.81	-0.88		
21-06-02	18:26:44	69.01	-167.36	17.44	-0.70		
21-06-02	19:26:44	68.99	-167.35	14.78	-0.73		
21-06-02	20:26:44	68.99	-167.37	15.95	-0.70		
21-06-02	21:26:44	68.89	-167.76	13.01	-0.71		
21-06-02	22:26:44	68.78	-168.14	15.66	-0.80		
21-06-02	23:26:44	68.69	-168.26	13.40	-0.87	5.02E-01	1.01E-01
21-06-03	00:26:44	68.52	-168.25	14.13	-0.64	1.06E-03	2.39E-04
21-06-03	01:26:44	68.41	-168.25	13.62	-0.77		
21-06-03	02:26:44	68.25	-168.25	13.73	-0.95		
21-06-03	03:26:44	68.14	-168.15	14.14	-0.88		
21-06-03	04:26:44	68.00	-168.00	12.98	-0.78	2.24E-01	4.24E-02
21-06-03	05:26:44	68.00	-168.00	14.38	-0.74	4.27E-04	9.92E-05
21-06-03	06:26:44	68.00	-168.00	13.60	-0.79	4.48E-01	9.29E-02
21-06-03	07:26:44	68.00	-168.00	11.94	-0.79	3.18E-05	5.09E-06
21-06-03	08:26:44	68.00	-168.01	12.65	-0.81	1.12E+00	2.02E-01
21-06-03	09:26:44	68.00	-168.00	11.90	-0.80		
21-06-03	10:26:44	68.00	-168.00	12.59	-0.74		
21-06-03	11:26:44	68.00	-168.00	11.89	-0.71		
21-06-03	12:26:44	68.00	-168.00	9.99	-0.85		
21-06-03	13:26:44	68.00	-168.00	10.50	-0.88		
21-06-03	14:26:44	68.00	-168.00	10.31	-0.93		
21-06-03	15:26:44	68.00	-168.00	8.94	-0.94	8.84E-05	7.89E-06
21-06-03	16:26:44	68.00	-168.00		-0.95		
21-06-03	17:26:44	68.00	-168.00	7.86	-0.95		
21-06-03	18:26:44	67.93	-168.00	6.82	-0.75		
21-06-03	19:26:44	67.77	-168.00	7.23	-0.81	1.57E-04	9.22E-06
21-06-03	20:26:44	67.71	-168.00	4.28	-0.79	2.55E-04	5.24E-06
21-06-03	21:26:44	67.59	-168.00	2.67	-0.76	7.40E-01	5.92E-03
21-06-03	22:26:44	67.50	-168.00	3.84	-0.58	3.67E-04	6.09E-06
21-06-03	23:26:44	67.41	-168.00	5.24	-0.51		
21-06-04	00:26:44	67.29	-168.00	4.55	-0.58		
21-06-04	01:26:44	67.21	-168.00	3.95	-0.59	1.47E-04	2.59E-06

21-06-04	02:26:44	67.11	-168.00	5.11	-0.46	8.77E-01	2.59E-02
21-06-04	04:26:44	67.00	-168.00	5.84	0.02	1.33E-03	5.19E-05
21-06-04	05:26:44	67.00	-168.00	5.98	-0.15		
21-06-04	06:26:44	67.00	-168.00	5.71	-0.22	1.70E-04	6.32E-06
21-06-04	07:26:44	67.00	-168.00	7.14	-0.33		
21-06-04	08:26:44	67.00	-168.00	6.84	-0.24		
21-06-04	09:26:44	67.00	-168.00	7.57	-0.39		
21-06-04	10:26:44	67.00	-168.01	5.63	-0.51		
21-06-04	11:26:44	67.00	-168.00	5.14	-0.66		
21-06-04	12:26:44	67.00	-168.00	6.63	-0.22		
21-06-04	13:26:44	67.00	-168.00	6.37	0.02	1.25E-04	5.83E-06
21-06-04	14:26:44	67.00	-168.00	6.85	-0.09		
21-06-04	15:26:44	67.00	-168.00	7.52	-0.25		
21-06-04	16:26:44	67.00	-168.00	7.92	-0.30	4.11E-04	2.93E-05
21-06-04	17:26:44	67.00	-167.99	6.72	-0.29	1.78E-03	9.16E-05
21-06-04	18:26:44	67.00	-167.99	5.90	-0.29	3.15E-04	1.25E-05
21-06-04	19:26:44	67.00	-167.64	6.67	-0.26		
21-06-04	20:26:44	67.00	-167.18	8.31	0.35		
21-06-04	21:26:44	67.01	-166.72	6.85	0.17	7.74E-01	4.18E-02
21-06-04	22:26:44	67.01	-166.25	5.30	-0.22		
21-06-04	23:26:44	67.01	-165.79	1.97	-0.08		
21-06-05	00:26:44	67.02	-165.42	3.75	-0.38		
21-06-05	01:26:44	67.15	-165.33	4.28	0.02	1.06E+00	2.22E-02
21-06-05	02:26:44	67.18	-165.21	5.51	0.01		
21-06-05	03:26:44	67.18	-165.06	4.25	-0.26	4.06E-01	8.35E-03
21-06-05	04:26:44	67.18	-165.04	3.70	-0.51		
21-06-05	05:26:44	67.19	-165.04	2.78	-0.45		
21-06-05	06:26:44	67.19	-165.04	3.40	-0.30	3.07E-01	4.03E-03
21-06-05	07:26:44	67.20	-165.03	3.44	-0.40	1.42E+00	1.90E-02
21-06-05	08:26:44	67.21	-165.16	3.00	0.13	9.95E-01	1.03E-02
21-06-05	09:26:44	67.21	-165.35	1.64	0.49	3.68E-06	1.14E-08
21-06-05	10:26:44	67.13	-165.74	0.76	0.21		
21-06-05	11:26:44	67.06	-166.14	1.88	0.21	3.78E-01	1.53E-03
21-06-05	12:26:44	67.00	-166.53	2.69	0.51	4.33E-04	3.63E-06
21-06-05	13:26:44	66.94	-166.93	4.33	0.46		
21-06-05	14:26:44	66.87	-167.33	5.94	0.12	1.27E-04	5.16E-06

21-06-05	15:26:44	66.79	-167.71	7.32	0.33		
21-06-05	16:26:44	66.75	-168.00	6.55	0.72	5.01E-01	2.51E-02
21-06-05	17:26:44	66.60	-167.99	6.79	0.55	5.79E-04	3.10E-05
21-06-05	18:26:44	66.50	-168.00	5.74	0.45	6.23E-01	2.38E-02
21-06-05	19:26:44	66.35	-168.00	6.74	0.30		
21-06-05	20:26:44	66.24	-168.00	7.66	0.17	7.84E-01	5.30E-02
21-06-05	21:26:44	66.09	-168.10	6.86	0.26	1.01E+00	5.46E-02
21-06-05	22:26:44	65.95	-168.19	6.56	0.10	5.63E-01	2.78E-02
21-06-05	23:26:44	65.80	-168.27	7.00	0.20	1.00E+00	5.65E-02
21-06-06	00:26:44	65.70	-168.32	3.66	0.18	1.98E-01	3.05E-03
21-06-06	01:26:44	65.56	-168.35	4.18	0.34	5.73E-01	1.16E-02
21-06-06	02:26:44	65.51	-168.36	3.85	0.45	6.07E-01	1.05E-02
21-06-06	03:26:44	65.53	-168.38	4.36	0.40	7.75E-01	1.71E-02
21-06-06	04:26:44	65.51	-168.36	3.43	0.31		
21-06-06	05:26:44	65.37	-168.26	3.45	0.44	1.14E+00	1.57E-02
21-06-06	06:26:44	65.23	-168.15	2.08	0.40	4.83E-01	2.42E-03
21-06-06	07:26:44	65.07	-168.05	1.08	0.46	1.54E+00	2.09E-03
21-06-06	08:26:44	64.99	-167.99	1.03	0.57	4.36E-01	5.37E-04
21-06-06	09:26:44	64.82	-167.99	1.71	0.73	1.19E+00	4.07E-03
21-06-06	10:26:44	64.72	-168.00	1.11	0.97	7.08E-01	1.02E-03
21-06-06	11:26:44	64.55	-168.00	3.76	0.70	5.61E-01	9.26E-03
21-06-06	12:26:44	64.44	-168.00	3.88	1.01	2.65E-01	4.70E-03
21-06-06	13:26:44	64.28	-168.00	2.55	0.53	7.68E-01	5.81E-03
21-06-06	14:26:44	64.11	-167.99	3.25	0.11	7.45E-01	9.05E-03
21-06-06	15:26:44	63.95	-167.96	4.06	-0.55	9.12E-01	1.70E-02
21-06-06	16:26:44	63.84	-167.69	3.16	0.20		
21-06-06	17:26:44	63.76	-167.36	5.22	1.92		
21-06-06	18:26:44	63.63	-167.23	4.75	1.30	6.54E-04	1.75E-05
21-06-06	19:26:44	63.50	-167.19	5.21	1.32	1.49E-04	4.79E-06
21-06-06	20:26:44	63.34	-167.22	7.29	4.50	2.87E-01	1.97E-02
21-06-06	21:26:44	63.18	-167.27	7.93	4.33	4.12E-04	3.32E-05
21-06-06	22:26:44	63.01	-167.35	8.26	4.98		
21-06-06	23:26:44	63.00	-167.37	6.68	5.05	7.56E-05	4.42E-06
21-06-07	00:26:44	63.00	-167.37	7.95	5.03		
21-06-07	01:26:44	62.96	-167.42	7.90	5.07	5.41E-01	4.41E-02
21-06-07	02:26:44	62.82	-167.59	6.16	5.04	5.31E-04	2.63E-05

21-06-07	03:26:44	62.68	-167.76	4.44	5.38	1.91E-04	4.97E-06
21-06-07	04:26:44	62.55	-167.94	4.23	5.35	9.05E-05	2.13E-06
21-06-07	05:26:44	62.46	-168.00	5.07	4.59	9.66E-05	3.21E-06
21-06-07	06:26:44	62.29	-168.00	5.31	4.22		
21-06-07	07:26:44	62.13	-168.00	4.28	4.11	7.69E-05	1.80E-06
21-06-07	08:26:44	61.96	-168.00	4.20	2.34	8.16E-01	1.75E-02
21-06-07	09:26:44	61.80	-168.00	1.59	1.43		
21-06-07	10:26:44	61.63	-168.00	4.52	2.46	2.25E-04	5.61E-06
21-06-07	11:26:44	61.50	-168.00	6.10	2.30		
21-06-07	12:26:44	61.38	-168.15	7.64	2.17	3.25E-01	2.30E-02
21-06-07	13:26:44	61.25	-168.32	7.84	1.89	1.20E-03	8.87E-05
21-06-07	14:26:44	61.12	-168.46	8.49	1.98		
21-06-07	15:26:44	61.01	-168.58	9.29	1.94		
21-06-07	16:26:44	60.91	-168.72	9.41	1.86	2.97E-04	3.17E-05
21-06-07	17:26:44	60.83	-168.83	8.93	2.17	6.29E-04	6.09E-05
21-06-07	18:26:44	60.83	-168.81	7.85	2.18	5.80E-04	4.34E-05
21-06-07	19:26:44	60.78	-168.79	10.40	2.22	7.14E-04	9.38E-05
21-06-07	20:26:44	60.62	-168.71	11.11	2.67	8.74E-01	1.33E-01
21-06-07	21:26:44	60.50	-168.64	10.13	2.82	2.35E-04	2.98E-05
21-06-07	22:26:44	60.36	-168.58	10.72	2.91		
21-06-07	23:26:44	60.20	-168.50	8.48	2.75	4.14E-04	3.67E-05
21-06-08	00:26:44	60.05	-168.42	9.34	2.97	3.05E-04	3.30E-05
21-06-08	01:26:44	59.91	-168.35		3.04	3.02E-04	
21-06-08	02:26:44	59.76	-168.30	6.83	3.07		
21-06-08	03:26:44	59.61	-168.23	5.61	3.04		
21-06-08	04:26:44	59.45	-168.15	5.48	3.03	1.63E+00	6.07E-02
21-06-08	05:26:44	59.29	-168.09	4.56	3.04	5.98E-04	1.55E-05
21-06-08	06:26:44	59.12	-168.01	3.25	3.20	7.11E-04	9.38E-06
21-06-08	07:26:44	58.95	-167.93	3.41	3.11	1.42E-04	2.05E-06
21-06-08	08:26:44	58.77	-167.86	3.09	3.19	6.23E-04	7.40E-06
21-06-08	09:26:44	58.60	-167.78	3.19	3.37		
21-06-08	10:26:44	58.42	-167.71	5.72	3.58	5.06E-01	2.09E-02
21-06-08	11:26:44	58.26	-167.63	7.09	3.81		
21-06-08	12:26:44	58.09	-167.55	10.30	4.65		
21-06-08	13:26:44	57.92	-167.48	12.37	4.57	6.17E-04	1.22E-04
21-06-08	14:26:44	57.76	-167.41	11.51	4.87	5.82E-04	1.00E-04

21-06-08	15:26:44	57.60	-167.34	11.24	5.15	1.87E-01	3.09E-02
21-06-08	16:26:44	57.45	-167.27	10.36	5.41	2.22E-04	3.14E-05
21-06-08	17:26:44	57.29	-167.20	9.03	5.45	6.59E-01	7.11E-02
21-06-08	18:26:44	57.14	-167.14	9.27	5.55		
21-06-08	19:26:44	56.98	-167.08	9.04	5.60	6.84E-01	7.42E-02
21-06-08	20:26:44	56.83	-167.00	8.55	5.76	2.31E-01	2.25E-02
21-06-08	21:26:44	56.67	-166.93	8.37	6.09	5.86E-01	5.52E-02
21-06-08	22:26:44	56.52	-166.87	7.95	6.32	5.65E-01	4.82E-02
21-06-08	23:26:44	56.36	-166.80	5.08	6.29	1.60E+00	5.58E-02
21-06-09	00:26:44	56.20	-166.74	4.35	6.50	1.00E+00	2.58E-02
21-06-09	01:26:44	56.04	-166.67	6.68	6.61	2.24E+00	1.36E-01
21-06-09	02:26:44	55.87	-166.60	6.22	6.59	1.55E+00	8.14E-02
21-06-09	03:26:44	55.70	-166.53	7.37	6.61	3.67E+00	2.71E-01
21-06-09	04:26:44	55.53	-166.48	8.11	6.74	1.99E+00	1.79E-01
21-06-09	05:26:44	55.35	-166.39	9.86	6.52	5.29E+00	6.99E-01
21-06-09	06:26:44	55.16	-166.32	12.01	6.48	3.72E+00	7.29E-01
21-06-09	07:26:44	54.99	-166.25	12.72	6.33	1.19E+01	2.60E+00
21-06-09	08:26:44	54.83	-166.19	11.77	6.25	8.86E+00	1.65E+00
21-06-09	09:26:44	54.68	-166.12	11.98	6.18	1.43E+01	2.76E+00
21-06-09	10:26:44	54.52	-166.05	13.93	6.05	1.15E+01	2.99E+00
21-06-09	11:26:44	54.46	-166.02	13.01	6.01	4.13E+01	9.38E+00
21-06-09	12:26:44	54.47	-166.04	11.39	6.03	2.54E+01	4.41E+00
21-06-09	13:26:44	54.47	-166.04	13.70	6.02	3.58E+01	9.00E+00
21-06-09	14:26:44	54.46	-166.04	11.55	6.03	2.28E+01	4.07E+00
21-06-09	15:26:44	54.45	-166.03	11.75	6.04	3.27E+01	6.06E+00
21-06-09	16:26:44	54.47	-166.04	10.72	5.99	2.17E+01	3.34E+00
21-06-09	17:26:44	54.47	-166.04	11.30	5.92	3.13E+01	5.34E+00
21-06-09	18:26:44	54.47	-166.04	11.60	6.01	1.63E+01	2.93E+00
21-06-09	19:26:44	54.47	-166.04	9.79	6.06	2.13E+01	2.74E+00
21-06-09	20:26:44	54.46	-166.04	9.17	6.06	1.25E+01	1.41E+00
21-06-09	21:26:44	54.47	-166.04	9.55	6.02	1.95E+01	2.38E+00
21-06-09	22:26:44	54.47	-166.04	10.00	6.03	1.21E+01	1.63E+00
21-06-09	23:26:44	54.46	-166.04	10.36	6.05	1.95E+01	2.81E+00
21-06-10	00:26:44	54.43	-165.91	11.92	6.28	1.15E+01	2.20E+00
21-06-10	01:26:44	54.38	-165.62	9.53	6.22	3.95E+01	4.83E+00
21-06-10	02:26:44	54.35	-165.34	7.37	6.01	1.63E+01	1.18E+00

21-06-10	03:26:44	54.32	-165.07	8.61	6.07	1.77E+01	1.76E+00
21-06-10	04:26:44	54.31	-164.84	10.48	5.93	8.81E+00	1.29E+00
21-06-10	05:26:44	54.28	-164.58	11.96	6.54	7.33E+00	1.42E+00
21-06-10	06:26:44	54.25	-164.28	8.40	6.60	9.98E-01	9.58E-02
21-06-10	07:26:44	54.22	-163.98	9.92	6.66	2.65E+00	3.54E-01
21-06-10	08:26:44	54.20	-163.68	7.38	6.60	1.82E+00	1.35E-01
21-06-10	09:26:44	54.18	-163.38	7.20	6.89	4.13E+00	2.93E-01
21-06-10	10:26:44	54.15	-163.09	8.36	6.80	2.17E+00	2.08E-01
21-06-10	11:26:44	54.13	-162.78	7.05	7.03	2.82E+00	1.93E-01
21-06-10	12:26:44	54.10	-162.48	2.54	7.02	1.53E+00	1.36E-02
21-06-10	13:26:44	54.11	-162.20	4.71	6.58	2.67E+00	8.05E-02
21-06-10	14:26:44	54.13	-161.91	7.79	6.59	2.08E+00	1.72E-01
21-06-10	15:26:44	54.15	-161.62	5.77	6.62	4.02E+00	1.82E-01
21-06-10	16:26:44	54.17	-161.33	3.94	6.89	2.74E+00	5.83E-02
21-06-10	17:26:44	54.17	-161.20	4.40	7.29	3.37E+00	9.01E-02
21-06-10	18:26:44	54.15	-161.23	5.30	7.29	2.16E+00	8.40E-02
21-06-10	19:26:44	54.17	-161.02	5.07	7.24	3.83E+00	1.36E-01
21-06-10	20:26:44	54.20	-160.79	5.56	7.61	1.90E+00	8.22E-02
21-06-10	21:26:44	54.23	-160.54	5.24	7.71	2.85E+00	1.09E-01
21-06-10	22:26:44	54.26	-160.30	6.41	7.62	2.12E+00	1.22E-01
21-06-10	23:26:44	54.28	-160.05	4.88	7.78	3.15E+00	1.05E-01
21-06-11	00:26:44	54.29	-159.81	4.67	8.14	1.60E+00	4.96E-02
21-06-11	01:26:44	54.32	-159.58	4.75	8.22	2.48E+00	7.93E-02
21-06-11	02:26:44	54.34	-159.35	3.78	8.42	1.19E+00	2.42E-02
21-06-11	03:26:44	54.38	-159.12	4.09	8.12	1.76E+00	4.16E-02
21-06-11	04:26:44	54.47	-158.92	3.13	8.26	1.27E+00	1.78E-02
21-06-11	05:26:44	54.55	-158.71	2.90	8.37	2.14E+00	2.57E-02
21-06-11	06:26:44	54.65	-158.51	3.97	8.30	1.30E+00	2.92E-02
21-06-11	07:26:44	54.75	-158.32	4.60	8.22	1.84E+00	5.54E-02
21-06-11	08:26:44	54.85	-158.11	6.77	7.96	9.20E-01	5.93E-02
21-06-11	09:26:44	54.94	-157.90	8.65	7.60	2.09E+00	2.18E-01
21-06-11	10:26:44	55.03	-157.67	9.25	7.66	1.63E+00	1.95E-01
21-06-11	11:26:44	55.10	-157.50	10.87	8.20	2.42E+00	4.06E-01
21-06-11	12:26:44	55.18	-157.32	9.33	7.91	1.64E+00	2.01E-01
21-06-11	13:26:44	55.27	-157.11	9.92	7.66	4.18E+00	5.74E-01
21-06-11	14:26:44	55.34	-156.90	7.88	7.65		

21-06-11	15:26:44	55.41	-156.71	7.74	7.26		
21-06-11	19:10:47	55.59	-156.36	5.43	8.06	3.85E+00	1.60E-01
21-06-11	20:10:47	55.71	-156.52	4.90	7.48	4.92E+00	1.64E-01
21-06-11	21:10:47	55.85	-156.69	2.38	7.74	2.82E+00	2.24E-02
21-06-11	22:10:47	55.99	-156.88	1.99	7.00	4.01E+00	2.18E-02
21-06-11	23:10:47	56.05	-156.96	2.13	7.66	2.66E+00	1.68E-02
21-06-12	00:10:47	56.11	-156.94	3.10	6.95	4.01E+00	5.28E-02
21-06-12	01:10:47	56.24	-156.86	3.89	7.58	2.74E+00	5.80E-02
21-06-12	02:10:47	56.36	-156.74	5.96	8.24	2.66E+00	1.34E-01
21-06-12	03:07:33	56.46	-156.61	7.07	8.74	2.33E+00	1.67E-01
21-06-12	05:16:19	56.67	-156.31	8.70	8.17	1.97E+00	2.11E-01
21-06-12	06:16:19	56.76	-156.16	6.58	7.92	3.40E+00	2.07E-01
21-06-12	07:16:19	56.84	-156.00	6.42	8.08	2.04E+00	1.18E-01
21-06-12	08:16:19	56.97	-155.83	0.82	8.49	3.32E+00	3.21E-03
21-06-12	09:16:19	57.10	-155.64	1.64	8.48	2.03E+00	7.81E-03
21-06-12	10:16:19	57.23	-155.43	7.87	8.03	2.84E+00	2.48E-01
21-06-12	11:16:19	57.35	-155.22	5.13	7.91	2.77E+00	1.02E-01
21-06-12	12:16:19	57.47	-155.01	5.38	7.82	3.92E+00	1.59E-01
21-06-12	13:16:19	57.59	-154.80	7.62	7.65	3.23E+00	2.62E-01
21-06-12	14:16:19	57.69	-154.56	6.63	8.10	4.25E+00	2.64E-01
21-06-12	15:16:19	57.73	-154.30	12.73	7.98	3.78E+00	8.63E-01
21-06-12	16:16:19	57.69	-154.01	3.19	8.93	4.31E+00	6.33E-02
21-06-12	17:16:19	57.59	-153.90	1.77	9.04	3.10E+00	1.40E-02
21-06-12	18:16:19	57.59	-153.90	4.76	8.89	3.59E+00	1.17E-01
21-06-12	19:16:19	57.59	-153.90	4.61	8.98	2.84E+00	8.76E-02
21-06-12	20:16:19	57.59	-153.90	4.92	9.09	3.61E+00	1.26E-01
21-06-12	21:16:19	57.59	-153.89	6.66	8.95	2.84E+00	1.82E-01
21-06-12	22:16:19	57.58	-153.89	5.71	8.95	3.54E+00	1.67E-01
21-06-12	23:16:19	57.58	-153.89	4.65	8.99	2.43E+00	7.60E-02
21-06-13	00:16:19	57.66	-153.95	4.65	8.66	3.50E+00	1.09E-01
21-06-13	01:16:19	57.78	-154.04	9.26	8.68	2.93E+00	3.61E-01
21-06-13	02:16:19	57.90	-153.96	7.46	8.19	4.09E+00	3.23E-01
21-06-13	03:16:19	58.02	-153.82	5.81	8.02	3.42E+00	1.63E-01
21-06-13	04:16:19	58.12	-153.64	5.61	8.27	4.29E+00	1.91E-01
21-06-13	05:16:19	58.23	-153.48	5.59	8.07	3.35E+00	1.48E-01
21-06-13	06:16:19	58.35	-153.31	6.25	8.43	5.22E+00	2.90E-01

21-06-13	07:16:19	58.46	-153.14	6.01	8.08	4.61E+00	2.35E-01
21-06-13	08:16:19	58.56	-152.99	5.04	8.29	3.03E+00	1.09E-01
21-06-13	09:16:19	58.67	-152.84	5.53	8.16	1.85E+00	7.99E-02
21-06-13	10:16:19	58.76	-152.67	7.65	7.82	2.96E+00	2.43E-01
21-06-13	11:16:19	58.77	-152.43	7.47	7.67	3.48E+00	2.72E-01
21-06-13	12:16:19	58.76	-152.21	6.66	8.03	1.35E+01	8.44E-01
21-06-13	13:16:19	58.78	-151.96	2.40	8.27	5.02E+00	4.10E-02
21-06-13	14:16:19	58.79	-151.70	3.02	7.96	3.92E+00	5.02E-02
21-06-13	15:16:19	58.83	-151.45	2.95	8.14	4.22E+00	5.21E-02
21-06-13	16:16:19	58.90	-151.21	4.00	8.03	4.47E+00	1.01E-01

Table S3.

Atmospheric particulate THg in the fine size aerosol measured by the Tekran speciation system during the Arctic cruise. Measured concentrations below the detection limitation (DL) are marked as '< DL'. Note: a) Date and Time are recorded in UTC time zone; b) Latitude in North and South denotes as '+' and '-'; c) Longitude in East and West denotes as '+' and '-'.

Date[a]	Time[a]	Lat[b]	Lon[c]	Hg^P (pg/m^3)	Date[a]	Time[a]	Lat[b]	Lon[c]	Hg^P (pg/m^3)
21-05-22	15:53:38	59.00	-173.00	< DL	21-06-04	01:35:00	67.19	-168.00	< DL
21-05-22	17:53:38	59.04	-172.97	< DL	21-06-04	03:35:00	67.00	-168.00	< DL
21-05-23	17:15:00	61.64	-168.64	< DL	21-06-04	07:35:00	67.00	-168.00	< DL
21-05-23	19:15:00	61.81	-168.42	< DL	21-06-04	09:35:00	67.00	-168.00	< DL
21-05-23	22:55:00	62.01	-168.59	1.03	21-06-04	12:15:00	67.01	-168.00	< DL
21-05-24	00:55:00	62.01	-168.62	< DL	21-06-04	14:15:00	67.00	-168.00	< DL
21-05-24	02:55:00	62.08	-168.44	< DL	21-06-04	16:35:00	67.00	-168.00	< DL
21-05-24	05:35:00	62.24	-168.02	< DL	21-06-04	20:35:00	67.00	-167.11	< DL
21-05-24	07:35:00	62.52	-167.83	1.67	21-06-04	22:35:00	67.01	-166.18	< DL
21-05-24	09:35:00	62.83	-167.64	2.60	21-06-05	00:35:00	67.04	-165.44	< DL
21-05-24	11:35:00	63.06	-167.51	2.05	21-06-05	02:35:00	67.18	-165.20	< DL
21-05-24	13:35:00	63.30	-167.48	< DL	21-06-05	10:35:00	67.12	-165.79	< DL
21-05-24	15:35:00	63.45	-167.45	1.03	21-06-05	12:35:00	66.99	-166.59	< DL
21-05-24	18:10:00	63.66	-167.20	0.98	21-06-05	15:15:00	66.81	-167.63	< DL
21-05-25	00:10:00	64.38	-167.24	1.27	21-06-05	19:35:00	66.33	-168.00	< DL
21-05-25	02:10:00	64.72	-167.39	1.19	21-06-05	21:35:00	66.07	-168.11	< DL
21-05-25	04:10:00	65.00	-167.68	< DL	21-06-05	23:35:00	65.78	-168.29	< DL
21-05-25	06:35:00	65.06	-167.67	< DL	21-06-06	01:35:00	65.54	-168.35	< DL
21-05-25	09:15:00	65.47	-167.97	< DL	21-06-06	07:35:00	65.05	-168.03	< DL
21-05-25	15:15:00	66.33	-168.29	< DL	21-06-06	11:35:00	64.53	-168.00	< DL
21-05-25	17:15:00	66.48	-167.66	< DL	21-06-06	13:35:00	64.26	-168.00	< DL
21-05-25	19:15:00	66.71	-167.45	< DL	21-06-06	15:35:00	63.93	-167.93	< DL
21-05-25	21:15:00	67.00	-167.42	< DL	21-06-06	20:15:00	63.37	-167.22	< DL
21-05-25	23:15:00	67.31	-167.40	< DL	21-06-06	22:15:00	63.04	-167.34	< DL
21-05-26	01:15:00	67.59	-167.85	1.49	21-06-07	00:15:00	63.00	-167.37	< DL
21-05-26	03:15:00	67.85	-168.27	2.88	21-06-07	03:22:50	62.69	-167.75	< DL
21-05-26	05:15:00	67.97	-168.42	3.38	21-06-07	05:22:50	62.47	-168.00	< DL
21-05-26	07:35:00	68.12	-168.41	2.58	21-06-07	07:22:50	62.14	-168.00	< DL
21-05-26	12:15:00	68.43	-168.56	2.39	21-06-07	09:22:50	61.81	-168.00	< DL
21-05-26	14:15:00	68.66	-168.19	1.53	21-06-07	11:22:50	61.50	-168.00	< DL

21-05-26	16:15:00	68.87	-167.66	< DL
21-05-26	20:15:00	69.00	-167.32	< DL
21-05-26	22:15:00	69.01	-167.31	< DL
21-05-27	00:15:00	69.25	-166.82	< DL
21-05-27	04:15:00	69.59	-165.81	< DL
21-05-27	06:15:00	69.89	-165.53	< DL
21-05-27	23:15:00	70.01	-166.15	< DL
21-05-28	18:15:00	70.05	-165.48	1.28
21-05-28	20:15:00	70.24	-164.76	< DL
21-05-28	22:15:00	70.38	-164.01	< DL
21-05-29	00:15:00	70.47	-164.00	< DL
21-05-29	06:15:00	70.45	-164.09	< DL
21-05-30	15:35:00	70.14	-165.03	< DL
21-05-31	04:15:00	70.10	-164.85	< DL
21-05-31	06:15:00	70.10	-164.90	< DL
21-05-31	08:15:00	70.10	-164.96	< DL
21-05-31	22:35:00	70.15	-165.42	1.05
21-06-01	00:35:00	70.14	-165.47	1.03
21-06-01	03:15:00	70.12	-165.53	1.22
21-06-01	09:15:00	69.75	-166.60	1.31
21-06-01	11:15:00	69.76	-166.62	< DL
21-06-01	13:30:00	69.76	-166.69	< DL
21-06-02	03:30:00	69.69	-167.01	1.19
21-06-02	06:05:00	69.73	-165.98	1.18
21-06-02	08:05:00	69.66	-165.84	1.50
21-06-02	10:05:00	69.42	-166.51	2.50
21-06-02	14:35:00	69.33	-167.80	< DL
21-06-02	20:35:00	68.98	-167.42	3.78
21-06-03	06:35:00	68.00	-168.00	2.06
21-06-03	09:15:00	68.00	-168.00	1.53
21-06-03	11:15:00	68.00	-168.00	1.53
21-06-03	13:15:00	68.00	-168.00	< DL
21-06-03	15:35:00	68.00	-168.00	< DL
21-06-03	17:35:00	68.00	-168.00	< DL
21-06-03	21:35:00	67.57	-168.00	< DL

21-06-07	13:22:50	61.26	-168.31	< DL
21-06-07	15:22:50	61.02	-168.57	< DL
21-06-07	17:22:50	60.83	-168.83	< DL
21-06-07	19:45:00	60.73	-168.76	< DL
21-06-07	22:25:00	60.37	-168.58	< DL
21-06-08	00:25:00	60.06	-168.42	< DL
21-06-08	02:25:00	59.76	-168.30	< DL
21-06-08	04:25:00	59.46	-168.15	< DL
21-06-08	06:25:00	59.13	-168.01	< DL
21-06-08	08:25:00	58.78	-167.86	< DL
21-06-08	10:25:00	58.43	-167.71	< DL
21-06-08	12:25:00	58.09	-167.55	< DL
21-06-09	05:20:00	55.37	-166.40	< DL
21-06-09	07:20:00	55.01	-166.26	< DL
21-06-09	09:20:00	54.69	-166.13	< DL
21-06-09	11:20:00	54.45	-166.01	< DL
21-06-09	13:20:00	54.47	-166.04	< DL
21-06-09	15:20:00	54.45	-166.03	< DL
21-06-09	17:20:00	54.47	-166.04	< DL
21-06-09	21:40:00	54.47	-166.04	< DL
21-06-09	23:40:00	54.46	-166.04	1.35
21-06-11	02:40:00	54.35	-159.29	< DL
21-06-11	04:40:00	54.49	-158.88	< DL
21-06-11	07:20:00	54.74	-158.34	< DL
21-06-11	09:20:0	54.93	-157.92	< DL
21-06-11	11:20:00	55.09	-157.52	< DL
21-06-11	13:20:00	55.26	-157.13	< DL
21-06-11	15:20:00	55.41	-156.73	< DL
21-06-11	17:20:00	55.58	-156.38	< DL
21-06-11	19:20:00	55.60	-156.38	< DL
21-06-11	21:20:00	55.87	-156.72	< DL
21-06-12	10:20:00	57.24	-155.42	< DL
21-06-12	12:20:00	57.48	-155.00	< DL
21-06-12	18:20:00	57.59	-153.90	7.19
21-06-12	20:20:00	57.59	-153.90	2.56

Table S4.

Atmospheric Hg species concentration in the bulk aerosol collected by the high-volume aerosol sampler during the Arctic cruise. The detailed information of each deployment and aerosol results from a previous nearby Arctic cruise are included(*27*). Note: a) Date and Time are recorded in UTC time zone; b) Latitude in North and South denotes as '+' and '-'; c) Longitude in East and West denotes as '+' and '-'.

Cruise	Sample ID	Date[a] Str	Lat[b] Str	Lon[c] Str	Date[a] End	Lat[b] End	Lon[c] End	Conc MeHg (pg/m^3)	STD Conc MeHg (pg/m^3)	Conc THg (pg/m^3)	STD Conc THg (pg/m^3)	MeHg (%)	STD MeHg (%)
This	01009	20210521	55.92	-169.35	20210524	63.76	-167.18	0.006396	0.000795	0.2776	0.0240	2.30	0.35
This	02009	20210524	63.76	-167.18	20210526	68.95	-167.47	0.015007	0.002004	0.9723	0.2144	1.54	0.40
This	05009	20210526	68.97	-167.41	20210529	70.46	-164.02	0.002315	0.000210	0.1776	0.0498	1.30	0.38
This	07009	20210529	70.46	-164.03	20210531	70.16	-165.35	0.001919	0.000763	0.4388	0.0249	0.44	0.18
This	08009	20210531	70.16	-165.36	20210604	67.00	-168.00	0.013579	0.001069	0.8324	0.0933	1.63	0.22
This	11009	20210604	67.00	-168.00	20210607	63.00	-167.37	0.000611	0.000236	0.0272	0.0047	2.25	0.95
This	14009	20210607	63.00	-167.37	20210609	54.47	-166.04	0.001012	0.000135	0.0237	0.0021	4.27	0.69
This	16009	20210609	54.47	-166.04	20210612	57.59	-153.90	0.005618	0.000235	0.4197	0.0164	1.34	0.08
(*27*)		8/10/15	56.07	170.51	8/17/15	69.93	-167.69	0.003082	0.000775	0.8351	0.1246	0.37	
(*27*)	12317	10/7/15	72.00	162.56	10/9/15	65.95	-168.45	0.001283		0.2608	0.0439	0.49	
(*27*)	12310	10/3/15	73.40	156.77	10/7/15	72.00	-162.56	0.001283		0.4079	0.1667	0.31	
(*27*)	12181	9/29/15	75.06	150.21	10/3/15	73.43	-156.79	0.001321		0.1719	0.0761	0.77	

Table S5.

Hg species in the rainwater collected by the N-CON rain sampler during the Arctic cruise. The detailed information of the rain event and rainwater results from a previous nearby Arctic cruise are included(*27*). Note: a) Date and Time are recorded in UTC time zone; b) Latitude in North and South denotes as '+' and '-'; c) Longitude in East and West denotes as '+' and '-'.

Cruise	Sample ID	Date[a] Str	Time[a] Str	Date[a] End	Time[a] End	Lat[b]	Lon[c]	Conc MeHg (pM)	Conc THg (pM)	STD Conc THg (pM)	MeHg (%)	STD MeHg (%)
This Study	16010	20210608	20:00	20210609	22:05	56.90	-167.04	0.4194	5.4714	1.4465	7.67	2.17
(*27*)	6041	8/14/15	3:25	8/16/15	6:49	63.78	170.94	0.0240	1.1464		2.09	
(*27*)	6108	8/16/15	9:06	8/21/15	22:04	66.02	168.73	0.0080	0.9262		0.86	
(*27*)	6172	8/21/15	23:41	8/31/15	21:24	81.94	176.99	0.0100	1.2568		0.80	
(*27*)	6496	10/5/15	20:05	10/6/15	18:35	72.42	161.18	0.1160	5.2598		2.21	

Table S6.

Hg species in the snow collected over sea-ice during the Arctic cruise. Note: a) Date and Time are recorded in UTC time zone; b) Latitude in North and South denotes as '+' and '-'; c) Longitude in East and West denotes as '+' and '-'.

Cruise	Sample ID	Date[a]	Time[a]	Station	Lat[b]	Lon[c]	Conc MeHg (pM)	STD Conc MeHg (pM)	Conc THg (pM)	STD Conc THg (pM)	MeHg (%)	STD MeHg (%)
This Study	S5-Snow1	20210528	23:22	5	70.02	-166.13	0.151	0.022	4.29	1.33	3.51	1.20
This Study	S6-Snow1	20210530	2:00	6	70.39	-164.20	0.108	0.008	4.34	0.13	2.48	0.20
This Study	S6-Snow2	20210530	2:00	6	70.39	-164.20	0.076	0.008	5.05	0.27	1.50	0.18
This Study	S6-Snow3	20210530	2:00	6	70.39	-164.20	0.095	0.015	4.87	0.16	1.95	0.30
This Study	S6-Snow4	20210530	2:00	6	70.39	-164.20	0.126	0.014	2.62	0.14	4.79	0.59
This Study	S9-Snow1	20210602	12:00	9	69.75	-166.96	0.124	0.019	10.55	0.07	1.18	0.18
This Study	S9-Snow2	20210602	12:00	9	69.75	-166.96	0.228	0.019	16.77	1.52	1.36	0.17
This Study	S9-Snow3	20210602	12:00	9	69.75	-166.96	0.016	0.013	3.13	0.18	0.50	0.41

Table S7.

Hg species in the surface seawater collected by the trace metal clean rosette during the Arctic cruise. Measured concentrations below the detection limitation (DL) are marked as '< DL'. The estimated DL for DMHg in surface seawater is 0.1 pg/L. Note: a) Date are recorded in UTC time zone; b) Latitude in North and South denotes as '+' and '-'; c) Longitude in East and West denotes as '+' and '-'.

Date[a]	Lat[b]	Lon[c]	Station	Depth (m)	Conc MeHg (pg/L)	STD Conc MeHg (pg/L)	Conc THg (ng/L)	STD Conc THg (ng/L)	Conc DMHg (pg/L)
20210522	59.00	-173.00	1	10			0.0345	0.0012	1.10
20210523	62.01	-168.56	2	7	0.99	0.41	0.1500	0.0015	0.32
20210525	65.01	-167.69	3	10			0.2633	0.0020	< DL
20210526	69.00	-167.34	4	10	1.64	0.63	0.1347	0.0008	1.19
20210527	70.02	-166.13	5	10	9.21	0.21	0.1925	0.0006	0.73
20210529	70.39	-164.20	6	10	7.63	0.28	0.1516	0.0005	0.38
20210530	70.14	-165.06	7	10	8.64	0.25	0.1636	0.0004	0.67
20210601	70.14	-165.48	8	10	3.96	1.08	0.1580	0.0019	< DL
20210601	69.75	-166.96	9	10	5.91	0.41	0.2032	0.0051	< DL
20210602	68.99	-167.36	10	38	0.88	0.42	0.1968	0.0017	< DL
20210603	68.00	-168.00	11	10	5.42	1.06	0.2126	0.0015	< DL
20210604	67.00	-167.99	12	12	2.46	0.39	0.3336	0.0044	< DL
20210606	65.51	-168.37	13	10	7.54	0.53	0.1551	0.0047	0.85
20210606	63.00	-167.37	14	9	4.51	0.77	0.2744	0.0033	< DL
20210607	60.83	-168.82	15	10	0.31	0.01	0.1938	0.0053	0.96
20210609	54.46	-166.04	16	10	363.85	23.82	0.4998	0.0024	7.22

Table S8.

Air-sea exchange of MeHg and DMHg in the polar marine boundary including DMHg evasion flux and wet and dry deposition fluxes of MeHg. Note: a) Measured DMHg concentrations below detection limitation are included in the uncertainty calculation of DMHg flux.

	Coastal Alaska	South Bering	North Bering	Chukchi Sea
DMHg Evasion Flux (pmol/m^2/hr)[a]	1.59 ± 2.21	0.14 ± 0.17	0.03 ± 0.06	0.03 ± 0.05
Wet Deposition MeHg (pmol/m^2/hr)	1.111 ± 0.096	0.046 ± 0.004	0.034 ± 0.003	0.047 ± 0.005
Dry Deposition MeHg (pmol/m^2/hr)	0.002 ± 0.0001	0.001 ± 0.0001	0.005 ± 0.0003	0.005 ± 0.0002
Net Flux (pmol/m^2/hr)	0.48 ± 2.21	0.09 ± 0.17	-0.01 ± 0.06	-0.02 ± 0.05

REFERENCES AND NOTES

1. R. P. Mason, W. F. Fitzgerald, Alkylmercury species in the equatorial Pacific. *Nature* **347**, 457–459 (1990).

2. C. T. Driscoll, R. P. Mason, H. M. Chan, D. J. Jacob, N. Pirrone, Mercury as a global pollutant: Sources, pathways, and effects. *Environ. Sci. Technol.* **47**, 4967–4983 (2013).

3. R. P. Mason, A. L. Choi, W. F. Fitzgerald, C. R. Hammerschmidt, C. H. Lamborg, A. L. Soerensen, E. M. Sunderland, Mercury biogeochemical cycling in the ocean and policy implications. *Environ. Res.* **119**, 101–117 (2012).

4. C.-S. Lee, N. S. Fisher, Methylmercury uptake by diverse marine phytoplankton. *Limnol. Oceanogr.* **61**, 1626–1639 (2016).

5. AMAP/UNEP, "Technical background report to the global mercury assessment 2018" (Arctic Monitoring and Assessment Programme, Oslo, Norway/UNEP Chemicals Branch Geneva, Switzerland, 2018).

6. K. L. Bowman, C. H. Lamborg, A. M. Agather, A global perspective on mercury cycling in the ocean. *Sci. Total Environ.* **710**, 136166 (2020).

7. C. H. Conaway, F. J. Black, M. Gault-Ringold, J. T. Pennington, F. P. Chavez, A. R. Flegal, Dimethylmercury in coastal upwelling waters, Monterey Bay, California. *Environ. Sci. Technol.* **43**, 1305–1309 (2009).

8. A. M. Agather, K. L. Bowman, C. H. Lamborg, C. R. Hammerschmidt, Distribution of mercury species in the Western Arctic Ocean (U.S. GEOTRACES GN01). *Mar. Chem.* **216**, 103686 (2019).

9. K. L. Bowman, C. R. Hammerschmidt, C. H. Lamborg, G. J. Swarr, A. M. Agather, Distribution of mercury species across a zonal section of the eastern tropical South Pacific Ocean (U.S. GEOTRACES GP16). *Mar. Chem.* **186**, 156–166 (2016).

10. A. T. Schartup, A. L. Soerensen, L.-E. Heimbürger-Boavida, Influence of the Arctic sea-ice regime shift on sea-ice methylated mercury trends. *Environ. Sci. Technol. Lett.* **7**, 708–713 (2020).

11. S. W. Effler, N. S. Bloom, Seasonal variability in the Mercury speciation of Onondaga Lake (New York). *Water Air Soil Pollut.* **53**, 251–265 (1990).

12. P. Quevauviller, O. F. X. Donard, J. C. Wasserman, F. M. Martin, J. Schneider, Occurrence of methylated tin and dimethyl mercury compounds in a mangrove core from Sepetiba Bay, Brazil. *Appl. Organomet. Chem.* **6**, 221–228 (1992).

13. J. H. Weber, R. Evans, S. H. Jones, M. E. Hines, Conversion of mercury(II) into mercury(0), monomethylmercury cation, and dimethylmercury in saltmarsh sediment slurries. *Chemosphere* **36**, 1669–1687 (1998).

14. I. Lehnherr, V. L. St. Louis, H. Hintelmann, J. L. Kirk, Methylation of inorganic mercury in polar marine waters. *Nat. Geosci.* **4**, 298–302 (2011).

15. R. P. Mason, K. A. Sullivan, The distribution and speciation of mercury in the South and equatorial Atlantic. *Deep Sea Res. Part II Top. Stud. Oceanogr.* **46**, 937–956 (1999).

16. R. P. Mason, "The chemistry of mercury in the equatorial Pacific Ocean," thesis, University of Connecticut (1991).

17. H. M. Adams, X. Cui, C. H. Lamborg, A. T. Schartup, Dimethylmercury as a source of monomethylmercury in a highly productive upwelling system. *Environ. Sci. Technol.* **58**, 10591–10600 (2024).

18. J. West, S. Gindorf, S. Jonsson, Photochemical degradation of dimethylmercury in natural waters. *Environ. Sci. Technol.* **56**, 5920–5928 (2022).

19. P. S. Weiss-Penzias, M. S. Bank, D. L. Clifford, A. Torregrosa, B. Zheng, W. Lin, C. C. Wilmers, Marine fog inputs appear to increase methylmercury bioaccumulation in a coastal terrestrial food web. *Sci. Rep.* **9**, 17611 (2019).

20. K. H. Coale, W. A. Heim, J. Negrey, P. Weiss-Penzias, D. Fernandez, A. Olson, H. Chiswell, A. Byington, A. Bonnema, S. Martenuk, A. Newman, C. Beebe, C. Till, The distribution and speciation of mercury in the California current: Implications for mercury transport via fog to land. *Deep Sea Res II Top. Stud. Oceanogr.* **151**, 77–88 (2018).

21. P. M. Outridge, R. P. Mason, F. Wang, S. Guerrero, L. E. Heimbürger-Boavida, Updated global and oceanic mercury budgets for the United Nations Global Mercury Assessment 2018. *Environ. Sci. Technol.* **52**, 11466–11477 (2018).

22. Y. Zhang, P. Zhang, Z. Song, S. Huang, T. Yuan, P. Wu, V. Shah, M. Liu, L. Chen, X. Wang, J. Zhou, Y. Agnan, An updated global mercury budget from a coupled atmosphere-land-ocean model: 40% more re-emissions buffer the effect of primary emission reductions. *One Earth* **6**, 316–325 (2023).

23. S. Jonsson, M. N. Mastromonaco, F. Wang, A. G. Bravo, W. R. L. Cairns, J. Chételat, T. A. Douglas, G. Lescord, L. Ukonmaanaho, L.-E. Heimbürger-Boavida, Arctic methylmercury cycling. *Sci. Total Environ.* **850**, 157445 (2022).

24. A. L. Soerensen, D. J. Jacob, A. T. Schartup, J. A. Fisher, I. Lehnherr, V. L. St. Louis, L.-E. Heimbürger, J. E. Sonke, D. P. Krabbenhoft, E. M. Sunderland, A mass budget for mercury and methylmercury in the Arctic Ocean. *Global Biogeochem. Cycles* **30**, 560–575 (2016).

25. P. A. Baya, M. Gosselin, I. Lehnherr, V. L. St Louis, H. Hintelmann, Determination of monomethylmercury and dimethylmercury in the Arctic marine boundary layer. *Environ. Sci. Technol.* **49**, 223–232 (2015).

26. A. Dastoor, H. Angot, J. Bieser, J. H. Christensen, T. A. Douglas, L.-E. Heimbürger-Boavida, M. Jiskra, R. P. Mason, D. S. McLagan, D. Obrist, P. M. Outridge, M. V. Petrova, A. Ryjkov, K. A. St. Pierre, A. T. Schartup, A. L. Soerensen, K. Toyota, O. Travnikov, S. J. Wilson, C. Zdanowicz, Arctic mercury cycling. *Nat. Rev. Earth Environ.* **3**, 270–286 (2022).

27. B. P. DiMento, R. P. Mason, S. Brooks, C. Moore, The impact of sea ice on the air-sea exchange of mercury in the Arctic Ocean. *Deep Sea Res I Oceanogr. Res. Pap.* **144**, 28–38 (2019).

28. V. L. St. Louis, H. Hintelmann, J. A. Graydon, J. L. Kirk, J. Barker, B. Dimock, M. J. Sharp, I. Lehnherr, Methylated mercury species in Canadian High Arctic marine surface waters and snowpacks. *Environ. Sci. Technol.* **41**, 6433–6441 (2007).

29. I. Lehnherr, V. L. St. Louis, C. A. Emmerton, J. D. Barker, J. L. Kirk, Methylmercury cycling in High Arctic wetland ponds: Sources and sinks. *Environ. Sci. Technol.* **46**, 10514–10522 (2012).

30. R. P. Mason, C. R. Hammerschmidt, C. H. Lamborg, K. L. Bowman, G. J. Swarr, R. U. Shelley, The air-sea exchange of mercury in the low latitude Pacific and Atlantic Oceans. *Deep Sea Res I Oceanogr. Res. Pap.* **122**, 17–28 (2017).

31. R. P. Mason, W. F. Fitzgerald, G. M. Vandal, The sources and composition of mercury in Pacific Ocean rain. *J. Atmos. Chem.* **14**, 489–500 (1992).

32. C. H. Conaway, F. J. Black, P. Weiss-Penzias, M. Gault-Ringold, A. R. Flegal, Mercury speciation in Pacific coastal rainwater, Monterey Bay, California. *Atmos. Environ.* **44**, 1788–1797 (2010).

33. P. Weiss-Penzias, K. Coale, W. Heim, D. Fernandez, A. Oliphant, C. Dodge, D. Hoskins, J. Farlin, R. Moranville, A. Olson, Total- and monomethyl-mercury and major ions in coastal California fog water: Results from two years of sampling on land and at sea. *Elementa* **4**, 000101 (2016).

34. R. Dietz, R. J. Letcher, J. P. Desforges, I. Eulaers, C. Sonne, S. Wilson, E. Andersen-Ranberg, N. Basu, B. D. Barst, J. O. Bustnes, J. Bytingsvik, T. M. Ciesielski, P. E. Drevnick, G. Gabrielsen, A. Haarr, K. Hylland, B. M. Jenssen, M. Levin, M. A. McKinney, R. D. Norregaard, K. E. Pedersen, J. Provencher, B. Styrishave, S. Tartu, J. Aars, J. T. Ackerman, A. Rosing-Asvid, R. Barrett, A. Bignert, E. W. Borns, M. Branigan, B. Braune, C. E. Bryan, M. Dam, C. A.

Eagles-Smith, M. Evans, T. J. Evans, A. T. Fisk, M. Gamberg, K. Gustavson, C. A. Hartman, B. Helander, M. P. Herzog, P. F. Hoekstra, M. Houde, K. Hoydal, A. K. Jackson, J. Kucklick, E. Lie, L. Loseto, M. L. Mallory, C. Miljeteig, A. Mosbech, D. C. G. Muir, S. T. Nielsen, E. Peacock, S. Pedro, S. H. Peterson, A. Polder, F. F. Rigét, P. Roach, H. Saunes, M. H. S. Sinding, J. U. Skaare, J. Sondergaard, G. Stenson, G. Stern, G. Treu, S. S. Schuur, G. Víkingsson, Current state of knowledge on biological effects from contaminants on arctic wildlife and fish. *Sci. Total Environ.* **696**, 133792 (2019).

35. N. Basu, M. Horvat, D. C. Evers, I. Zastenskaya, P. Weihe, J. Tempowski, A state-of-the-science review of mercury biomarkers in human populations worldwide between 2000 and 2018. *Environ. Health Perspect.* **126**, 106001 (2018).

36. R. S. D. Calder, S. Bromage, E. M. Sunderland, Risk tradeoffs associated with traditional food advisories for Labrador Inuit. *Environ. Res.* **168**, 496–506 (2019).

37. F. Pithan, T. Mauritsen, Arctic amplification dominated by temperature feedbacks in contemporary climate models. *Nat. Geosci.* **7**, 181–184 (2014).

38. K. A. St. Pierre, S. Zolkos, S. Shakil, S. E. Tank, V. L. St. Louis, S. V. Kokelj, Unprecedented increases in total and methyl mercury concentrations downstream of retrogressive thaw slumps in the western Canadian Arctic. *Environ. Sci. Technol.* **52**, 14099–14109 (2018).

39. K. Schaefer, Y. Elshorbany, E. Jafarov, P. F. Schuster, R. G. Striegl, K. P. Wickland, E. M. Sunderland, Potential impacts of mercury released from thawing permafrost. *Nat. Commun.* **11**, 4650 (2020).

40. K. A. St. Pierre, V. L. St. Louis, I. Lehnherr, A. S. Gardner, J. A. Serbu, C. A. Mortimer, D. C. G. Muir, J. A. Wiklund, D. Lemire, L. Szostek, C. Talbot, Drivers of mercury cycling in the rapidly changing glacierized watershed of the High Arctic's largest lake by volume (Lake Hazen, Nunavut, Canada). *Environ. Sci. Technol.* **53**, 1175–1185 (2019).

41. S. Jonsson, M. G. Nerentorp Mastromonaco, K. Gårdfeldt, R. P. Mason, Distribution of total mercury and methylated mercury species in Central Arctic Ocean water and ice. *Mar. Chem.* **242**, 104105 (2022).

42. Y. He, X. Shi, W. W. Huffman, C. H. Lamborg, R. P. Mason, Description of a dimethylmercury automatic analyzer for the high-resolution measurement of dissolved gaseous mercury species in surface ocean waters. *Environ. Sci. Technol.* **56**, 13076–13084 (2022).

43. K. M. Munson, C. H. Lamborg, G. J. Swarr, M. A. Saito, Mercury species concentrations and fluxes in the Central Tropical Pacific Ocean. *Global Biogeochem. Cycles* **29**, 656–676 (2015).

44. R. P. Mason, K. R. Rolfhus, W. F. Fitzgerald, Mercury in the North Atlantic. *Mar. Chem.* **61**, 37–53 (1998).

45. J. L. Kirk, V. L. St. Louis, H. Hintelmann, I. Lehnherr, B. Else, L. Poissant, Methylated mercury species in marine waters of the Canadian High and Sub Arctic. *Environ. Sci. Technol.* **42**, 8367–8373 (2008).

46. P. Lin, R. S. Pickart, T. J. Weingartner, H. L. Simmons, M. Itoh, T. Kikuchi, Formation and circulation of newly ventilated winter water in the western Beaufort Sea. *Prog. Oceanogr.* **216**, 103068 (2023).

47. R. S. Pickart, P. Lin, F. Bahr, L. T. McRaven, J. Huang, A. Pacini, K. R. Arrigo, C. J. Ashjian, C. Berchok, M. F. Baumgartner, K.-H. Cho, L. W. Cooper, S. L. Danielson, D. Dasher, A. Fuiwara, J. Gann, J. M. Grebmeier, J. He, T. Hirawake, M. Itoh, L. Juranek, T. Kikuchi, G. W. K. Moore, J. Napp, R. J. Nelson, S. Nishino, H. Statscewich, P. Stabeno, K. M. Stafford, H. Ueno, S. Vagle, T. J. Weingartner, B. Williams, S. Zimmermann, The Pacific water flow branches in the eastern Chukchi Sea. *Prog. Oceanogr.* **219**, 103169 (2023).

48. M. C. Despins, R. P. Mason, A. M. Aguilar-Islas, C. H. Lamborg, C. R. Hammerschmidt, S. E. Newell, Linked mercury methylation and nitrification across oxic subpolar regions. *Front. Environ. Chem.* **4**, 1109537 (2023).

49. Tekran, “Tekran model 2537a, model 1130 mercury speciation unit and model 1135 particulate mercury unit” (User Manuals, Toronto, Canada, 2005), p. 1135.

50. A. Steffen, T. Douglas, M. Amyot, P. Ariya, K. Aspmo, T. Berg, J. Bottenheim, S. Brooks, F. Cobbett, A. Dastoor, A. Dommergue, R. Ebinghaus, C. Ferrari, K. Gardfeldt, M. E. Goodsite, D. Lean, A. J. Poulain, C. Scherz, H. Skov, J. Sommar, C. Temme, A synthesis of atmospheric mercury depletion event chemistry in the atmosphere and snow. *Atmos. Chem. Phys.* **8**, 1445–1482 (2008).

51. J. West, D. Babi, A. Azaroff, S. Jonsson, Dimethylmercury in natural waters—Analytical and experimental considerations. *Limnol. Oceanogr. Methods.* **21**, 837–846 (2023).

52. K. Gårdfeldt, J. Munthe, D. Strömberg, O. Lindqvist, A kinetic study on the abiotic methylation of divalent mercury in the aqueous phase. *Sci. Total Environ.* **304**, 127–136 (2003).

53. Y. Talmi, R. E. Mesmer, Studies on vaporization and halogen decomposition of methyl mercury compounds using gc with a microwave detector. *Water Res.* **9**, 547–552 (1975).

54. Å. Iverfeldt, O. Lindqvist, Distribution equilibrium of methyl mercury chloride between water and air. *Atmos. Environ. (1967)* **16**, 2917–2925 (1982).

55. C. R. Hammerschmidt, C. H. Lamborg, W. F. Fitzgerald, Aqueous phase methylation as a potential source of methylmercury in wet deposition. *Atmos. Environ.* **41**, 1663–1668 (2007).

56. H. Hersbach, B. Bell, P. Berrisford, G. Biavati, A. Horányi, J. Muñoz Sabater, J. Nicolas, C. Peubey, R. Radu, I. Rozum, D. Schepers, A. Simmons, C. Soci, D. Dee, J.-N. Thépaut, “ERA5 hourly data on single levels from 1940 to present” (Copernicus Climate Change Service (C3S) Climate Data Store (CDS), 2023).

57. H. Niki, P. D. Maker, C. M. Savage, L. P. Breitenbach, A long-path Fourier transform infrared study of the kinetics and mechanism for the hydroxyl radical-initiated oxidation of dimethylmercury. *J. Phys. Chem.* **87**, 4978–4981 (1983).

58. H. Niki, P. S. Maker, C. M. Savage, L. P. Breitenbach, A Fourier-transform infrared study of the kinetics and mechanism of the reaction of atomic chlorine with dimethylmercury. *J. Phys. Chem.* **87**, 3722–3724 (1983).

59. J. Sommar, M. Hallquist, E. Ljungström, O. Lindqvist, On the gas phase reactions between volatile biogenic mercury species and the nitrate radical. *J. Atmos. Chem.* **27**, 233–247 (1997).

60. Y. Zhang, A. L. Soerensen, A. T. Schartup, E. M. Sunderland, A global model for methylmercury formation and uptake at the base of marine food webs. *Global Biogeochem. Cycles* **34**, e2019GB006348 (2020).

61. B. P. DiMento, R. P. Mason, Factors controlling the photochemical degradation of methylmercury in coastal and oceanic waters. *Mar. Chem.* **196**, 116–125 (2017).

62. H. Demarcq, Trends in primary production, sea surface temperature and wind in upwelling systems (1998–2007). *Prog. Oceanogr.* **83**, 376–385 (2009).

63. G. Cutter, P. Andersson, L. Codispoti, P. Croot, R. François, M. C. Lohan, H. Obata, M. Rutgers v. d. Loeff, “Sampling and sample-handling protocols for GEOTRACES Cruises” (GEOTRACES International Project Office, 2010).

64. M. E. Andersson, K. Gårdfeldt, I. Wängberg, A description of an automatic continuous equilibrium system for the measurement of dissolved gaseous mercury. *Anal. Bioanal. Chem.* **391**, 2277–2282 (2008).

65. M. E. Andersson, J. Sommar, K. Gårdfeldt, O. Lindqvist, Enhanced concentrations of dissolved gaseous mercury in the surface waters of the Arctic Ocean. *Mar. Chem.* **110**, 190–194 (2008).

66. R. P. Mason, N. Lawson, G.-R. Sheu, Mercury in the Atlantic Ocean: Factors controlling air–sea exchange of mercury and its distribution in the upper waters. *Deep Sea Res. Part II Top. Stud. Oceanogr.* **48**, 2829–2853 (2001).

67. F. J. G. Laurier, R. P. Mason, L. Whalin, S. Kato, Reactive gaseous mercury formation in the North Pacific Ocean's marine boundary layer: A potential role of halogen chemistry. *J. Geophys. Res. Atmos.* **108**, 2003JD003625 (2003).

68. Y. He, R. P. Mason, Comparison of reactive gaseous mercury measured by KCl-coated denuders and cation exchange membranes during the Pacific GEOTRACES GP15 expedition. *Atmos. Environ.* **244**, 117973 (2021).

69. M. S. Landis, R. K. Stevens, F. Schaedlich, E. M. Prestbo, Development and characterization of an annular denuder methodology for the measurement of divalent inorganic reactive gaseous mercury in ambient air. *Environ. Sci. Technol.* **36**, 3000–3009 (2002).

70. A. F. Stein, R. R. Draxler, G. D. Rolph, B. J. B. Stunder, M. D. Cohen, F. Ngan, NOAA's HYSPLIT atmospheric transport and dispersion modeling system. *Bull. Am. Meteorol. Soc.* **96**, 2059–2077 (2015).

71. K. M. Munson, D. Babi, C. H. Lamborg, Determination of monomethylmercury from seawater with ascorbic acid-assisted direct ethylation. *Limnol. Oceanogr. Methods.* **12**, 1–9 (2014).

72. N. Bloom, W. F. Fitzgerald, Determination of volatile mercury species at the picogram level by low-temperature gas chromatography with cold-vapor atomic fluorescence detection. *Anal. Chim. Acta* **208**, 151–161 (1988).

73. W. F. Fitzgerald, G. A. Gill, Subnanogram determination of mercury by two-stage gold amalgamation and gas phase detection applied to atmospheric analysis. *Anal. Chem.* **51**, 1714–1720 (1979).

74. U.S. Environmental Protection Agency, "Method 1631, Revision E: Mercury in water by oxidation, purge and trap, and cold vapor atomic fluorescence spectrometry" (US EPA Office of Science and Technology, Washington, D.C., 2002).

75. C. M. Marsay, D. Kadko, W. M. Landing, P. L. Morton, B. A. Summers, C. S. Buck, Concentrations, provenance and flux of aerosol trace elements during US GEOTRACES Western Arctic cruise GN01. *Chem. Geol.* **502**, 1–14 (2018).

76. R. Wanninkhof, Relationship between wind speed and gas exchange over the ocean. *J. Geophys. Res. Oceans* **97**, 7373–7382 (1992).

77. L. Zhang, Z. He, Technical Note: An empirical algorithm estimating dry deposition velocity of fine, coarse and giant particles. *Atmos. Chem. Phys.* **14**, 3729–3737 (2014).

78. L. J. Barrett, P. Vlahos, D. E. Hammond, R. P. Mason, Sediment-water fluxes of inorganic carbon and nutrients in the Pacific Arctic during the sea ice melt season. *Cont. Shelf Res.* **268**, 105116 (2023).

79. O. Lindqvist, H. Rodhe, Atmospheric mercury—A review. *Tellus B* **37**, 136–156 (1985).

80. P. D. Nightingale, G. Malin, C. S. Law, A. J. Watson, P. S. Liss, M. I. Liddicoat, J. Boutin, R. C. Upstill-Goddard, In situ evaluation of air-sea gas exchange parameterizations using novel conservative and volatile tracers. *Global Biogeochem. Cycles* **14**, 373–387 (2000).

81. J. D. Holmes, *Wind loading of structures* (CRC press, 2007).

82. L. Poissant, M. Amyot, M. Pilote, D. Lean, Mercury water−air exchange over the Upper St. Lawrence River and Lake Ontario. *Environ. Sci. Technol.* **34**, 3069–3078 (2000).

83. C. R. Wilke, P. Chang, Correlation of diffusion coefficients in dilute solutions. *AIChE J.* **1**, 264–270 (1955).

84. W. Hayduk, H. Laudie, Prediction of diffusion coefficients for nonelectrolytes in dilute aqueous solutions. *AIChE J.* **20**, 611–615 (1974).

85. N. T. Loux, Monitoring cyclical air/water elemental mercury exchange. *J. Environ. Monit.* **3**, 43–48 (2001).

86. A. L. Soerensen, H. Skov, D. J. Jacob, B. T. Soerensen, M. S. Johnson, Global concentrations of gaseous elemental mercury and reactive gaseous mercury in the marine boundary layer. *Environ. Sci. Technol.* **44**, 7425–7430 (2010).
